# [C–H···anion] interactions mediate the templation and anion binding properties of topologically non-trivial metal–organic structures in aqueous solutions†
†Electronic supplementary information (ESI) available: For general methods, further details of synthesis and characterization, anions binding, and DFT and PM6 calculations. CCDC 1409618. For ESI and crystallographic data in CIF or other electronic format see DOI: 10.1039/c5sc04246a


**DOI:** 10.1039/c5sc04246a

**Published:** 2016-02-12

**Authors:** Rana A. Bilbeisi, Thirumurugan Prakasam, Matteo Lusi, Roberto El Khoury, Carlos Platas-Iglesias, Loïc J. Charbonnière, John-Carl Olsen, Mourad Elhabiri, Ali Trabolsi

**Affiliations:** a New York University Abu Dhabi (NYUAD) , Experimental Research Building (C1), Saadiyat Island , Abu Dhabi , UAE . Email: ali.trabolsi@nyu.edu; b Centro de Investigaciones Científicas Avanzadas (CICA) and Departamento de Química Fundamental , Universidade de Coruña , Campus da Zapateira, Rúa da Fraga 10 , 15008 A Coruña , Spain; c Laboratoire d'Ingénierie Moléculaire Appliquée à l'Analyse , IPHC , UMR 7178 CNRS/UdS , ECPM , 25 rue Becquerel , 67087 Strasbourg , France; d School of Sciences , Indiana University Kokomo , Kokomo , IN 46904 , USA; e Laboratoire de Chimie Bioorganique et Médicinale , UMR 7509 CNRS/UdS , ECPM , 25 rue Becquerel , 67087 Strasbourg , France . Email: elhabiri@unistra.fr

## Abstract

Two synthetic approaches—temperature variation and anion templation—allowed for the selective formation of a [2]catenane (**[2]C^4+^**) or a trefoil knot (**TK^6+^**), or for the enhanced formation of a Solomon link (**SL^8+^**), all from a simple set of starting materials (Zn(ii) acetate, diformylpyridine (DFP) and a diamino-2,2′-bipyridine (DAB)) in mixed aqueous solutions. The catenane formed exclusively at 90 °C in a 1 : 1 mixed solvent of D_2_O and MeOD. In the presence of bromide ion as template, **TK^6+^** formed exclusively at 50 °C in the same solvent. In the solid state, **TK^6+^** hosts two bromide ions in its central cavity by forming six C_sp^2^_–H hydrogen bonds. In D_2_O, **TK^6+^**, which was originally prepared as a trifluoroacetate (TFA) salt, was found to exchange two TFA counterions for two monovalent anions of different sizes and shapes, which lodged within the knot's central cavity. In contrast to bromide, the larger triflate anion (CF_3_SO_3_^–^) promoted the formation of **SL^8+^**, which was characterized by ^1^H NMR spectroscopy and mass spectrometry. Two dimensional heteronuclear ^19^F-^1^H-HOSEY NMR experiments detected CH···F interactions inside the cavity of **SL^8+^**. Thus, the product distribution of this dynamic link forming system is sensitive to temperature and the size and shape of the anion template, and one of the products, **TK^6+^**, is capable of binding a variety of monovalent anions in D_2_O with high affinity (with log *β*_2_ values of 4 to 6 being typical).

## Abstract 2

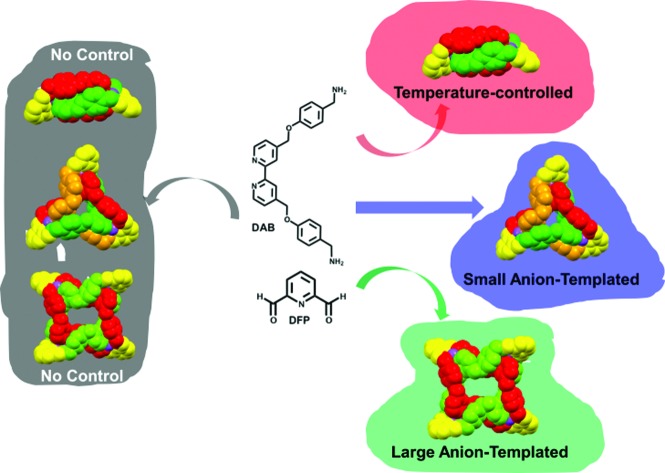
We report the anion-recognition properties and anion-mediated templation of Metal-Organic knots and links in aqueous solutions.

## Introduction

During the past 15 years, many small synthetic molecules displaying a variety of structural motifs have been developed for binding anions in organic or aqueous solvents.^1^–^6^ The most challenging goal, and the one with the greatest potential rewards in terms of practical applications, is the selective recognition of anions in water.^1^,^2^,^7^ Not surprisingly, a survey of natural anion receptors provides impressive benchmarks for comparison and emulation. For example, the sulfate-binding and transport protein of *Salmonella typhimurium* sequesters sulfate in water with a dissociation constant, *K*_D_, of 20 μM.^8^ The phosphate binding protein of *Escherichia coli* binds phosphate selectively with a *K*_D_ of 0.7 μM.^9^–^12^ Recent achievements involving the recognition of anions in water by synthetic receptors include the sensitive detection of pollutants,^13^–^18^ the transportation of ions across membranes, and the sensing of biologically relevant anions *in vivo*.^19^–^25^ Gale and co-workers have published comprehensive reviews of these applications and other recent highlights in the field.^26^–^28^

Previously, we reported a one-pot synthesis of a set of topologically non-trivial, Zn(ii)-templated complexes that were isolated as trifluoroacetate (TFA) salts: a [2]catenane, **[2]C(TFA)_4_**; a trefoil knot, **TK(TFA)_6_**, and a Solomon link, **SL(TFA)_8_**.^29^ By relying on reversible imine and metal–ligand bond formation (ref. 30) we were able to form all three complexes simultaneously from a simple pair of chelating ligands: diformylpyridine (DFP) and a diamino-2,2′-bipyridine (DAB).^30^ The [2]catenane was fully characterized by NMR spectroscopy, mass spectrometry and X-ray crystallography. The more complex structures, **TK(TFA)_6_** and **SL(TFA)_8_**, initially resisted full characterization. We could not grow X-ray quality crystals of **TK(TFA)_6_** and could only detect **SL** species by mass spectrometry at early stages of the reaction. We now report (i) the solid state characterization of a bromide containing trefoil knot complex, **TK(TFA)_4_Br_2_**, (ii) quantitative studies in D_2_O of the exchange of two TFA anions of the **TK(TFA)_6_** complex for various other monovalent anions and (iii) the effects of temperature and anion size and shape on the product distribution of the templation reaction itself. We demonstrate that different monovalent anions can be used to favor formation of either **TK^6+^** or **SL^8+^** in mixed aqueous solvents. A notable feature of this system is the cooperative effect of both cationic and anionic templates. The zinc(ii) cation is necessary for complex formation, whereas the anion template influences complex topology.

We would also like to call attention to the relatively rare structural motif by which **TK^6+^** and **SL^8+^** bind anions within their central cavities: multiple weak but cooperative C_sp^2^_–H hydrogen bonds. This motif is present in small anion binders such as the bisimidazoliums of the Maeda group,^31^ as well as the triazole-containing macrocycles and podands reported by Flood and coworkers.^32^–^34^ Previous examples of topologically interesting complexes in which this feature is present include Leigh's pentafoil knot,^35^–^37^ which is templated, in part, by a central chloride ion; and the chloride and nitrate-binding rotaxanes^38^,^39^ of the Beer group. Nevertheless, measurements of the anion binding affinities of molecular links and knots have rarely been reported.^38^,^40^ It is particularly remarkable that the C–H hydrogen bonding of the **TK^6+^** and **SL^8+^** complexes are effective in D_2_O, one of the most competitive solvents. We believe that the results described below, in particular, the anion binding studies of **TK^6+^** are a unique contribution to the field of aqueous anion receptor chemistry.

## Results & discussion

### Solid state structure of **TK^6+^**

**Fig. 1:**
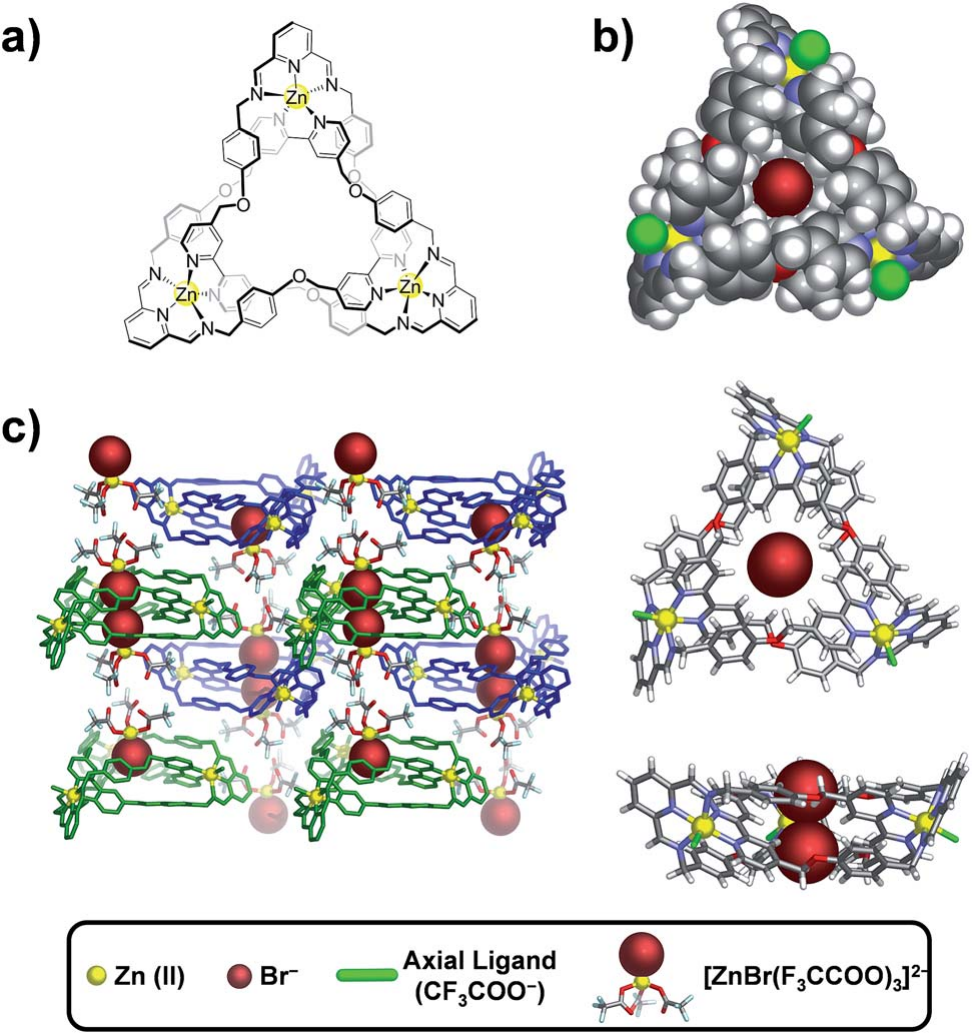
Single crystal structure of the Zn(ii)-based trefoil knot. (a) Molecular structure of **TK^6+^**. (b) Space-filling (top) and stick-figure (bottom) views of **[TK(TFA)_3_Br_2_]^+^**. (c) Crystal packing of **[TK(TFA)_3_]^3+^** and the adduct **[ZnBr(CF_3_COO)_3_]^2–^**.

Single crystals containing **TK(TFA)_4_Br_2_** and suitable for X-ray diffraction were isolated by slow vapor diffusion of *n*-butylether into a trifluoroethanol solution of **TK(TFA)_6_** that contained a small amount of tetrabutyl ammonium bromide. Tri-bladed propeller-shaped cationic complexes of **TK^6+^** crystallized as a racemic mixture in the trigonal *P*3[combining macron] space group. The crystal structure presented in Fig. 1b, depicts the *C*_3_ symmetry of the knotted Znii3L_3_ complex (where L represents the condensed DAB + DFP organic ligands). Three equivalent L strands are held together by three Zn(ii) ions located 13.3 Å apart. Each zinc cation is coordinated to five nitrogen atoms (two from the 2,2′-bipyridine and three from the 2,5-diiminopyridine) and has a distorted octahedral geometry, with its coordination sphere being completed by one trifluoroacetate anion. The bipyridines of the ligand strand are located between phenoxy substituents that are attached to the imine moieties. The shortest distance between phenoxy and bipyridyl rings is ∼3.5 Å, which is at the upper limit associated with effective π–π stacking interactions. An adduct composed of two tetrahedral **[ZnBr(CF_3_COO)_3_]^2–^** complexes that are hydrogen-bonded by water molecules is co-crystallized with the cationic knot (Fig. 1c). The presence of bromide anions was confirmed (Fig. S1†) by EDAX analysis of the single crystal used in the XRD experiment as well as by mass spectrometry in the gas phase. In the solid state, two bromide ions were found to occupy the central cavity of **TK^6+^** and seem to be essential for crystallization, as all attempts to crystallize **TK^6+^** from bromide-free solutions failed.

**TK^6+^** cations and adducts alternate regularly in such a way (Fig. 1c) that the bromides of two adducts reside in the center of **TK^6+^** and are each fixed by at least three CH···Br^–^ charge-assisted hydrogen bonds that range in length from 2.92 to 2.98 Å. The strength of binding between the **TK^6+^** cation and the adduct also likely facilitates crystallization of the knot.

The average diameter and average depth of the **TK^6+^** cavity, as deduced from the crystal structure, are 5.86 Å and 4.06 Å, respectively. In the solid state, this cylindrical pocket hosts two bromide anions by establishing CH···anion interactions.^32^,^41^–^42^

### Computational modeling of **TK^6+^**

Due to the large size of the **TK^6+^** complex, semi-empirical PM6 calculations were used to determine a theoretically optimal geometry. The PM6 algorithm had reproduced fairly well (see ESI†) the experimentally determined geometry of **[2]C^4+^**.^29^ Subsequent single-point calculations on the PM6-generated **TK^6+^** structure were performed using density functional theory (DFT) at the B3LYP/6-31G(d) level to determine the electrostatic potential at the surface of the knot. The electrostatic potential over the surface of **TK^6+^**, depicted in Fig. 3a, was calculated in aqueous solution as defined by an isodensity surface of 0.001 electrons bohr^–3^.^43^ Regions with the highest positive electrostatic potential are located on the aromatic C_sp^2^_–H hydrogens that point toward the center of the cavity.

The optimized geometry of **TK^6+^** presents a nearly undistorted *C*_3_ symmetry, where the symmetry axis passes through the center of the central cavity. However, the helical arrangement of the three pyridyl units of the supramolecular assembly results in a sizeable dipole moment directed along the *C*_3_ axis, which at the B3LYP/6-31G(d) level amounts to 2.77 D in the gas-phase and 4.97 D in aqueous solution (Fig. 3a).

Two centroids are defined by the three methylene protons that point to each side of the central cavity. These centroids delimit the cavity's height, which measures 6.57 Å. The radius of the base is 2.93 Å and is estimated from the distances between the methylene protons and the centroid that they define. Thus, assuming the simultaneous binding of two anions, the size of the **TK^6+^** cavity (∼130 Å^3^) is suited for the recognition of two anions of relatively small radius (≤2.5 Å).

The favorable distribution of positive charge in **TK^6+^** and its demonstrated ability to firmly host two bromide anions within its central cavity in the solid state inspired us to explore the knot's anion recognition properties in solution. There are few examples of molecular links that have pre-organized cavities capable of aromatic hydrogen bonding,^40^,^44^,^45^ and to the best of our knowledge quantitative anion binding studies involving knotted structures in aqueous media have not been reported.

**Fig. 2:**
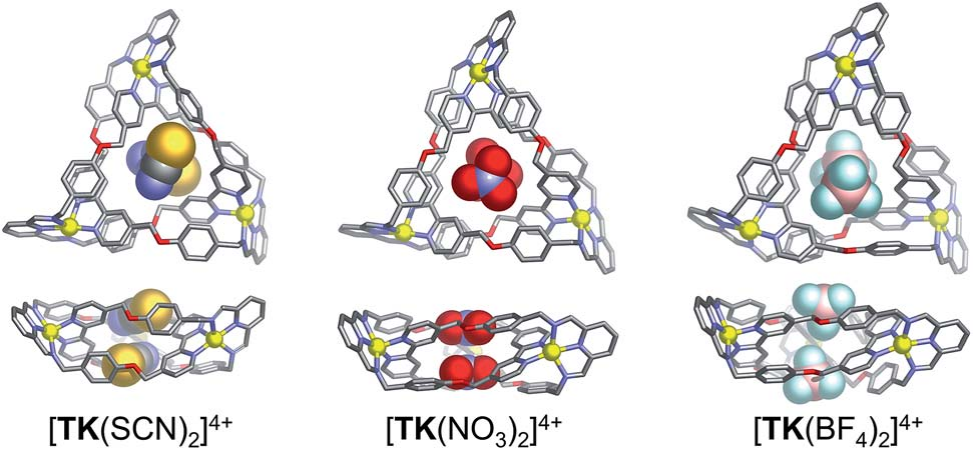
Top and side views of PM6-optimized geometries of host guest complexes involving **TK^6+^** and, from left to right, SCN^–^, NO_3_^–^ and BF_4_^–^.

The binding of monovalent anions of different size and shape to **TK^6+^** was initially evaluated computationally, using semi-empirical PM6 calculations. This computational study provided insight into possible binding modes and stoichiometries. The geometry optimized **[TKBr_2_]^4+^** complex (Fig. S2†) is in reasonably good agreement with the X-ray structure. Energy-minimized models of complexes of **TK^6+^** with linear (thiocyanate), trigonal planar (nitrate) and tetrahedral (tetrafluoroborate) anions are presented in Fig. 2. The anions are held within the knot's central cavity by multiple non-classical aromatic CH···anion hydrogen bonds.

### Measurement of anion exchange and binding

Initially, **TK^6+^** was prepared as its trifluoroacetate salt in isopropanol and characterized in methanol.^29^ After discovering that **TK(TFA)_6_** is soluble and stable in water, we designed experiments to assess the knot's anion binding ability in this highly competitive medium. Preliminary ^1^H-^19^F HOESY NMR spectroscopy experiments (SI) gave no indication of interactions between the fluorines of TFA and the hydrogens of the organic framework of **TK^6+^**; hence, the mode of association of TFA with **TK^6+^** could not be determined.

**Fig. 3:**
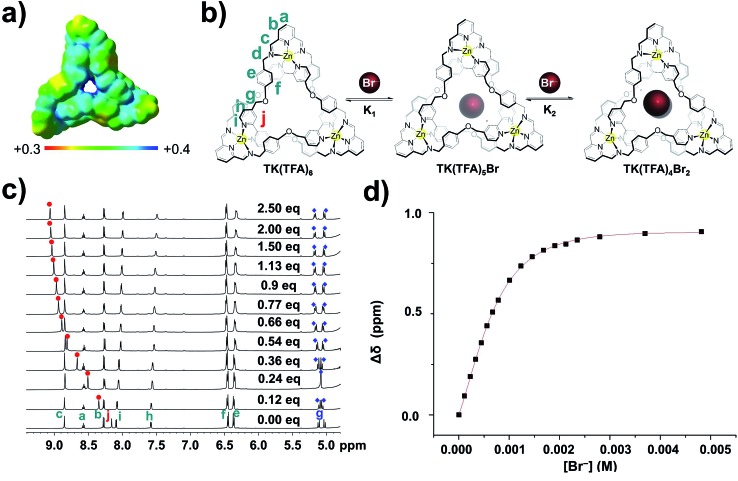
Measurement of the bromide ion binding constants of **TK^6+^** complexes by titration. (a) Computed B3LYP/6-31G(d) electrostatic potential of **TK^6+^** on the molecular surface defined by the 0.001 electrons bohr^–3^ contour of the electron density (b) schematic representation of bromide ion binding. (c) Stacked plots of ^1^H NMR (600 MHz, 298 K) spectra of 1.87 mM solutions of **TK^6+^** in D_2_O titrated with, bottom to top, increasing amounts of tetrabutylammonium bromide. (d) Binding isotherm obtained by plotting H_j_ signal shift *versus* bromide ion concentration. In most cases, addition of more than two equivalents of anion precipitated the knot and prevented further measurements.

The binding of bromide ion in water was evaluated in titration experiments monitored by ^1^H NMR spectroscopy. Incremental amounts of aqueous tetrabutylammonium bromide were added to a D_2_O solution of **TK(TFA)_6_** at room temperature. The spectra show (Fig. 3c) the gradual spectral shifts of **TK^6+^** resonances that occur as the result of the knot's interaction with bromide ions in solution. These continuous spectral changes are characteristic of an exchange process that is fast on the NMR timescale. The signal that corresponds to the H_j_ protons exhibits the largest downfield shift, from 8.18 ppm (before addition of Br^–^) to 9.10 ppm (upon saturation with the anion). Its change as a function of bromide ion concentration is illustrated in Fig. 3c. The signals that correspond to the diastereotopic H_g_ protons are significantly split apart as the concentration of bromide increases. The signal of the H_e_ protons displays a small shift, and the signals that correspond to the H_a_, H_b_, H_c_, and H_d_ protons are only slightly affected by bromide ion binding.

These spectroscopic results are consistent with the relative positions of the **TK^6+^** protons and bromide ions in the crystal structure of the knot, which shows, for example, that the H_a_, H_b_, H_c_, and H_d_ protons point away from the knot's cavity and that the H_g_ and H_j_ bipyridyl protons point toward its center and are involved in hydrogen bonding with bromide.

The shift data was processed and found to fit with good agreement to a 1 : 2 (**TK^6+^** : Br^–^) binding model, indicating that two bromides bind within the cavity of **TK^6+^** in D_2_O. (A 1 : 1 binding model, and others, were not consistent with the data.) Mass spectrometry experiments (see ESI†) provided strong evidence for the existence of the 1 : 2 complex in the gas phase. A series of *m*/*z* fragments corresponding to **[TK(TFA)_2_(Br)_2_]^2+^**, **[TK(TFA)_3_Br]^2+^**, and **[TK(TFA)_4_]^2+^** were detected by ESI-HRMS, with no evidence for complexes containing more than two bromide ions.

From the solid state and solution studies, we infer that two TFA anions located outside the **TK^6+^** cavity are exchanged for two bromides that lodge within the cavity. The process is facilitated by electrostatic attraction between the positively charged host cavity and the negatively charged bromides. Multiple CH···anion interactions are formed in the host–guest complex, even in the aqueous solvent. The process is dynamic, with bound bromides being exchanged continuously with bromides free in solution.

The first (*K*_1_) and second (*K*_2_) association constants (which can be considered to be binding constants) were calculated and found to be 4.4(0.5) × 10^2^ M^–1^ and 2.3(0.3) × 10^3^ M^–1^, respectively. Interestingly, the **TK(TFA)_4_Br_2_** complex is significantly more stable than the monobromide complex, **TK(TFA)_5_Br**, which suggests that the binding of two anions is a cooperative process. The ratio of the calculated binding constants (*K*_2_/*K*_1_) is approximately five which indicates positive cooperativity and suggests that binding of the first bromide ion causes conformational changes in the host's framework that facilitate binding of the second.^46^–^51^

The same general pattern of spectral shifts occurred during titrations of the knot with other monovalent anions of different size (ionic radii, *r*, of 1.7 to 2.4 Å) and shape (Fig. S3–S7†) including I^–^, N_3_^–^, SCN^–^, and NO_3_^–^ (Fig. S3–S6†).^1^,^32^,^52^

The pattern was somewhat different with BF_4_^–^ (Fig. S7†). The H_j_ signal was shifted, indicating an interaction between the anion and the interior surface of the knot's cavity, but the direction of the shift was upfield rather than downfield. A sample containing the knot and BF_4_^–^ was further analyzed in a ^1^H-^19^F HOESY experiment. The resulting spectrum (Fig. S8†) shows NOE interactions between the fluorine atoms of BF_4_^–^ and the H_g_ and H_j_ protons, which confirms the close proximity of the anion and the walls of the knot's central cavity. Thus, all of the selected anions, including BF_4_^–^, bind within the cavity, though BF_4_^–^ may extend beyond it, as suggested by computational modelling (Fig. 2, right).

The shift of the H_j_ signal that occurred during the titrations was used to calculate the association constants, *K*_1_ and *K*_2_, for all anions except BF_4_^–^, whose association constants were deduced from the shift of the H_h_ signal. A global binding constant, log *β*_2_, and a cooperativity parameter, *K*_2_/*K*_1_, were also calculated for each anion. The results are listed in Table 1. Regardless of size and shape, all of the selected anions were found to bind with the same 1 : 2 (**TK^6+^** : anion) stoichiometry. In the case of BF_4_^–^, this stoichiometry was supported by an ESI-HRMS analysis (see ESI†) that revealed a series of *m*/*z* fragments corresponding to the complexes **[TK(TFA)_2_(BF_4_)_2_]^2+^**, **[TK(TFA)_3_BF_4_]^2+^**, and **[TK(TFA)_4_]^2+^**, and no evidence for complexes involving more than two BF_4_^–^ anions.

**Table 1:** Successive (*K*_1_ and *K*_2_) and global (log *β*_2_) binding constants determined from ^1^H titrations of **TK^6^**^**+**^ with monovalent anions at room temperature in D_2_O. Binding constants were deduced from ^1^H chemical shifts measured as a function of anion concentration. H_j_ protons were monitored in all cases except the BF_4_^–^ titration, for which the H_h_ protons were monitored. Standard deviations are shown in parentheses

Anion	*K*_1_ (M^–1^)	*K*_2_ (M^–1^)	log *β*_2_	*K*_2_/*K*_1_
Br^–^	4.4(0.5) × 10^2^	2.3(0.3) × 10^3^	6.0	5.2(0.8)
I^–^	6.5(0.6) × 10^2^	2.2(0.3) × 10^3^	6.15	3.3(0.6)
N_3_^–^	9.8(1.7) × 10^2^	9.3(7.8) × 10^2^	5.96	1.0(0.8)
SCN^–^	1.5(0.3) × 10^2^	3.4(2.0) × 10^3^	5.72	22.5(2.5)
NO_3_^–^	5.2(0.2) × 10^2^	6.7(2.5) × 10^1^	4.54	0.13(0.05)
BF_4_^–^	1.0(0.2) × 10^3^	2.2(0.7) × 10^2^	5.34	0.21(0.08)

As compared to other small-molecule receptors, **TK^6+^** exhibits high affinities for the selected anions in water.^1^,^2^ However, the cooperativity of the two binding events associated with each anion type varied. For the spherical and linear anions, the *K*_2_/*K*_1_ ratio was always significantly larger (1.0 < *K*_2_/*K*_1_ < 23.0) than 0.25, indicating positive cooperativity.^51^ A comparison of the data for the two spherical anions investigated, Br^–^ (*r* = 1.82 Å) and I^–^ (*r* = 2.06 Å), revealed that I^–^ binds with a slightly higher global binding affinity, but with lower cooperativity (log *β*_2_ = 6.1, *K*_2_/*K*_1_ = 3.3), than Br^–^ (log *β*_2_ = 6.0, *K*_2_/*K*_1_ = 5.2). These results suggest that I^–^ fits better in the cylindrical cavity of **TK^6+^** but that the smaller size of Br^–^ minimizes anion–anion repulsion. Of the two linear anions, SCN^–^ (length = 2.13 Å) and N_3_^–^ (length = 1.71 Å), the larger one, SCN^–^, displays a comparable global binding constant but a higher cooperativity value (log *β*_2_ = 5.72, *K*_2_/*K*_1_ = 22.5) than N_3_^–^ (log *β*_2_ = 5.96, *K*_2_/*K*_1_ = 1.0). The unexpectedly higher cooperativity of SCN^–^ is consistent with the negative charge of this anion being more localized on the nitrogen atom, a property that leads to a more directed interaction with **TK^6+^** and results in structural adaptation that facilitates the binding of the second SCN^–^ anion. Considering the calculated structure of **[TK(SCN)_2_]^4+^** illustrated in Fig. 2, we hypothesize that the two SCN^–^ anions are bound in a staggered configuration that minimizes anion–anion repulsion and therefore increases binding affinity and cooperativity. Also, we suspect that resonance delocalization of the negative charge of N_3_^–^ likely weakens this anion's interaction with **TK^6+^** and fails to induce the conformational changes that would enhance binding cooperativity.

In contrast to the positively cooperative binding behavior of the spherical and linear anions, the binding of the larger trigonal planar (NO_3_^–^) and tetrahedral (BF_4_^–^) anions was negatively cooperative, with both *K*_2_/*K*_1_ values being lower than 0.25. In these cases, the first anion to bind hinders the second from binding due to increased steric and electronic repulsions. The *K*_2_/*K*_1_ ratio of 0.21 for BF_4_^–^ indicates statistically negative cooperation which can be attributed to the anion's relatively large ionic radius; however, BF_4_^–^ was found to bind to **TK^6+^** with relatively high overall affinity (log *β*_2_ = 5.34).

The larger trifluoromethylsulfonate (triflate, OTf^–^) anion was titrated as its tetrabutylammonium salt, and no signal shifting in the knot's ^1^H NMR spectrum was observed (Fig. S9†). This result indicates that OTf^–^ binding (to any part of the knot) is relatively weak under the experimental conditions and is likely due to the non-coordinating nature of the anion and its relatively large size which prevents entry into the knot's central cavity.

Binding experiments with Cl^–^, CN^–^, OCN^–^ and ClO_4_^–^ were also attempted, but all of these anions caused **TK^6+^** to precipitate from solution, which prevented accurate measurements. In addition to binding to the central cavity of **TK^6+^**, these anions might also be replacing TFA anions that coordinate axially to the Zn(ii) metal centers and in this way causing reduced solubility and precipitation of the complexes in aqueous media.

Using variable temperature NMR spectroscopy, we studied and compared the binding of Br^–^ with that of BF_4_^–^. At 298 K, the H_g_ and H_j_ signals are sharp in a spectrum (Fig. S15†) of a solution of the knot and Br^–^ measured at 298 K, which indicates that exchange of Br^–^ is relatively fast at room temperature. At 268 K, the H_g_ and H_j_ peaks are considerably broader, which reflects a slower exchange with respect to room temperature. In contrast, at 298 K, the H_g_ and H_j_ signals are broad in a spectrum (Fig. S16†) of a solution containing BF_4_^–^ and the knot, whereas they are sharp when the temperature is 333 K. Thus, for any given temperature, exchange of BF_4_^–^ is slower.

### Controlling **[2]C^4+^**, **TK^6+^** and **SL^8+^** populations in solution

The trifluoroacetate salt of the DAB ligand that was previously used^29^ for the synthesis of the three links was replaced by the corresponding neutral DAB molecule (see ESI† for synthetic details). Use of the neutral ligand prevented precipitation of the complexes in the mixed aqueous solvent and allowed us to monitor their simultaneous formation under different reaction conditions.^53^

Mixing neutral DAB with DFP and zinc(ii) acetate in a 1 : 1, D_2_O : MeOD solvent mixture, at temperatures ranging from 50 °C to 90 °C, lead to the formation of **TK^6+^** and **[2]C^4+^** in various proportions. When the reaction was carried out at 90 °C, **[2]C^4+^** was formed exclusively, whereas, at 50 °C, a significant amount (63%) of **TK^6+^** was formed. We found that the **TK^6+^** : **[2]C^4+^** ratio was dependent on both temperature and solvent. There were no signs of **SL^8+^** formation under any of these conditions. The fact that a greater proportion of **[2]C^4+^** formed at higher temperatures substantiates previous findings^29^ of ours that suggested that **[2]C^4+^** is the thermodynamic product of the reaction and that **TK^6+^** is a kinetic product.

We had postulated that bromide was responsible for significant stabilization of the X-ray structure of the knot. This hypothesis was supported by an NMR investigation of the ability of Br^–^ to template the formation of **TK^6+^** in solution. In the absence of a Br^–^ template, DFP, DAB and Zn(OAc)_2_ afforded a mixture of 63% **TK(TFA)_4_Br_2_** and 37% **[2]C(TFA)_4_** in a 1 : 1, D_2_O : MeOD solution at 50 °C. However, the mole fraction of the knot increased to 77% upon addition of one equivalent (relative to the stoichiometry of the starting materials) of tetrabutylammonium bromide, and addition of two equivalents of bromide resulted in an even greater proportion (85%) of the knot (Fig. S19†).

**Fig. 4:**
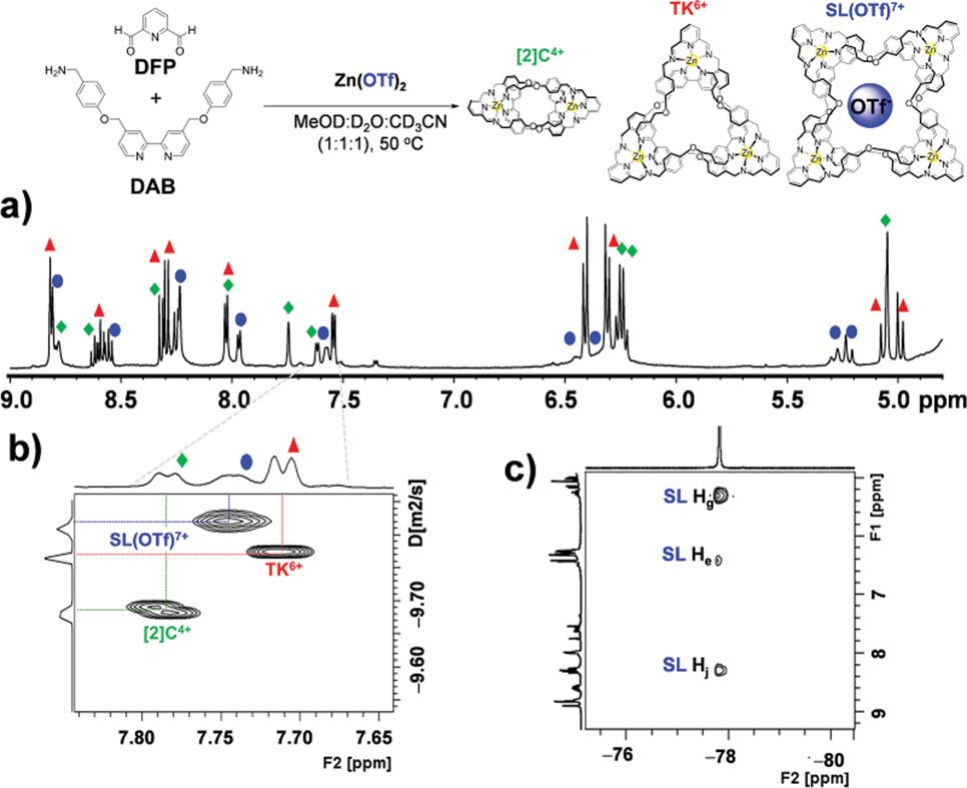
NMR spectroscopic evidence for the formation of **SL^8+^**. (a) ^1^H NMR, (b) DOSY and (c) HOESY spectra of a solution (MeOD : D_2_O : CD_3_CN, 1 : 1 : 1) of **[2]C^4+^**, **TK^6+^** and **[SL(OTf)]^7+^** at 500 MHz and 298 K.

In a 1 : 1 : 1 mixture of CD_3_OD, D_2_O and CD_3_CN the bulky triflate anion gave rise to an additional set of resonances in the spectrum of the reaction mixture (Fig. 4) that matched neither those of **TK^6+^** nor **[2]C^4+^**. Further NMR and mass spectrometric analysis allowed us to assign the new set of peaks to **SL^8+^** (Fig. S20†). **SL^8+^** is much larger than **TK^6+^** and has a large central cavity that may be able to accommodate the triflate anion. The size of the cavity was estimated with the aid of PM6 calculations. The optimized geometry of **SL^8+^** (Fig. S21†) presents a nearly undistorted *C*_2_ symmetry with a cylindrical cavity having a height of ∼5.90 Å (6.57 Å for the **TK^6+^**) and bases of *ca.* 4.52 Å radius (*versus* 2.39 Å for **TK^6+^**). Thus, the central cavity of **SL^8+^** is significantly wider than that of **TK^6+^**.

The ESI-HRMS spectrum of the reaction mixture in which triflate was used as a templating anion confirmed the presence of the three links. In addition to peaks characteristic of the catenane and the trefoil knot, the spectrum reveals two major peaks (Fig. S20†) with maxima at *m*/*z* 1036.44 and 1629.13, which are consistent with the cations **[SL(OTf)_5_]^3+^** (calculated *m*/*z* = 1036.45) and **[SL(OTf)_6_]^2+^** (calculated *m*/*z* = 1629.16).

Diffusion ordered ^1^H NMR spectroscopy (DOSY) of reaction mixtures that included OTf^–^ as a template produced spectra (Fig. 4b) that confirmed the presence of the three complexes. The diffusion coefficients of **[2]C^4+^**, **TK^6+^** and **SL^8+^** complexes were found to be 1.98(0.01) × 10^–10^ m^2^ s^–1^, 1.62(0.01) × 10^–10^ m^2^ s^–1^ and 1.460(0.005) × 10^–10^ m^2^ s^–1^, respectively. The corresponding hydrodynamic radii of the complexes were calculated to be 1.35, 1.65 and 1.83 nm, respectively (see ESI† for calculations). Moreover, ^1^H-^19^F heteronuclear NOESY (HOESY) experiments revealed through-space interactions between the aromatic protons of **SL^8+^**'s cavity and the fluorine nuclei of the triflate anion. Fig. 4c shows three cross peaks that indicate correlations between the fluorines and the H_e_, H_g_ and H_j_ protons of the Solomon link. These correlations confirm the close proximity of the protons and fluorines and provide evidence for triflate's role as a template. The H_g_ and H_j_ protons seem to be involved in stronger coupling interactions, as indicated by the greater signal intensities of their cross peaks. PM6 calculations performed on the **SL(OTf)^7+^** system provided (Fig. S21†) an optimized geometry in which triflate is held inside the cavity of the host by CH···O and CH···F interactions involving H_g_ and H_j_, and H_e_ protons, respectively, and which is in good qualitative agreement with the experimental measurements.

## Conclusions

Anion templation and temperature variation were used to control the product distribution of a dynamic library of zinc(ii)-based molecular knots and links (**[2]C^4+^**, **TK^6+^** and **SL^8+^**). Electrostatic forces, including weak non-covalent CH···anion interactions that operated in the MeOD/D_2_O solvent mixtures mediated the topological outcome of the reaction. In the solid state, the electropositive central cavity of **TK^6+^** was found to accommodate two bromide anions with multiple CH hydrogen-bonds. These CH···anion interactions occur between bipyridinyl units and bromide anions and are a major stabilizing feature in the packed crystal. In D_2_O, **TK^6+^** preserved its anion binding properties: monovalent anions of various shapes and sizes were found to bind to **TK^6+^** in 1 : 2 (**TK^6+^** : anion) stoichiometries and with high affinities, with log *β*_2_ values typically in the range of 4 to 6.

Thermodynamic control over the library's product distribution was possible by varying the temperature of the reaction and/or by changing the anion template. Catenane **[2]C^4+^**, being the most thermodynamically stable complex, was formed exclusively when the reaction was carried at 90 °C. Lowering the temperature to 50 °C caused **[2]C^4+^** and **TK^6+^** to form simultaneously in 37% and 63% chemical yields, respectively. Addition of two equivalents of bromide ion to the reaction at 50 °C resulted in a much greater proportion of **TK^6+^** (85%). Addition of the bulkier triflate anion (OTf^–^) allowed for formation and characterization of a Solomon link, **SL^8+^**. The presence of CH···F interactions inside the cavity of the **SL^8+^** were supported by 2D heteronuclear ^19^F-^1^H-HOSEY NMR experiments.

An analogous system in which both cations and anions influence the distribution of several metallosupramolecular products by templation has been described by Nitschke and coworkers.^54^ In that system a set of cages, helicates and prisms were formed. To our knowledge, ours is the first such library involving knots and links.

With further development, anion binding within the topologically unique cavities of these molecular complexes could find application in areas such as anion-sensing and anion-assisted catalysis. For example, incorporation of fluorogenic substituents into **TK^6+^** could allow for the sensing of specific anions.^41^ Furthermore, it might be possible to fabricate ion selective electrodes by modifying the surfaces of the electrodes with molecular knots and links.

## Supplementary Material

Crystal structure data

Supplementary information
