# Supplementary material for: [C–H···anion] interactions mediate the templation and anion binding properties of topologically non-trivial metal–organic structures in aqueous solutions
Source: Chem Sci. 2016 Feb 12;7(4):2524–31. doi: 10.1039/c5sc04246a (PMC5477011; doi:10.1039/c5sc04246a)
Supplement: Supplementary file 2 [file SC-007-C5SC04246A-s002.pdf]

Electronic Supplementary Material (ESI) for Chemical Science.
This journal is © The Royal Society of Chemistry 2016

Supporting Information

[C–H···Anion] Interactions Mediate the Templation and Anion Binding Properties of Topologically Non-trivial Metal-Organic Structures in Aqueous Solutions

Rana A. Bilbeisi,[1] Thirumurugan Prakasam,[1] Matteo Lusi,[1] Roberto El-Khoury,[1] Carlos Platas-Iglesias,[2]

Loïc J. Charbonnière,[3] John-Carl Olsen,[4] Mourad Elhabiri,[5]* and Ali Trabolsi[1]*

Table of Contents:

1.0 General **2**

2.0 X-ray Crystallography **2**

3.0 Theoretical calculations **8**

4.0 Anion recognition studies of TK(TFA)$_6$ **9**

4.1 *Iodide* 10

4.2 *Azide* 11

4.3 *Thiocycanate* 12

4.4 *Nitrate* 13

4.5 *Tetrafluoroborate* 14

4.6 *Trifluoromethanesulfonate* 15

4.7 HRMS-ESI of a 1 : 2 mixture of TK : Anion in water 16

4.8 Speciation diagrams of the host-guest complexes of TK with various anions 18

*4.9 Variable temperature ^{1}H NMR of TK(TFA)6 in presence of Br$^-$ and BF$_4^-$ and ^{19}F – ^{1}H HOESY NMR of TK(TFA)$_6$*19

5.0 Synthesis of neutral DAB **21**

6.0 Controlling the population of [2]C^{4+}, TK^{6+}, and SL^{8+} in solution **21**

6.1 *[2]Catenane* 21

6.2 *Trefoil knot* 22

6.3 *Solomon link* 23

6.4 *Hydrodynamic volume calculations* 25

The diffusion coefficient D is related to the hydrodynamic radius, r, of a molecule[12] by the equation: 25

7.0 References **26**

1.0 General

All reagents and starting materials were purchased from Sigma-Aldrich and used without further purification. The diamino bipyridine (DAB) ligand and 2,6-diformylpyridine (DFP) were synthesized as reported earlier.[1] Thin-layer chromatography (TLC) was performed on silica gel 60 F254 (E. Merck), and the developed plates were inspected by UV illumination. Column chromatography was performed on silica gel 60F (Merck 9385, 0.040–0.063 mm). Routine nuclear magnetic resonance (NMR) spectra were recorded at 25 °C on a Bruker Avance 600 spectrometer, with working frequencies of 600 MHz for ^{1}H, and 151.0 MHz for ^{13}C nuclei, respectively. All chemical shifts are reported in ppm relative to the signals corresponding to the residual non-deuterated solvents (CD_3CN: $\delta = 1.94$ ppm, CD_3OD: $\delta = 3.31$ ppm).[2] All ^{13}C spectra were recorded with the simultaneous decoupling of proton nuclei. Coupling constants (*J*) are given in hertz (Hz) with multiplicity abbreviated as follows: s (singlet), d (doublet), dd (doublet of doublets), t (triplet), q (quartet), qt (quintet), sx (sextet), m (multiplet). A wide signal is preceded by br (broad). High resolution mass spectrometric (HRMS) analyses were performed using an Agilent 6540 UHA Accurate Mass Q-TOF / LC - MS-spectrometer in the positive mode with an acetonitrile/water eluent gradient on a C-18 column.

2.0 X-ray Crystallography

Crystal structures were determined at 100 K by X-ray diffraction on a Bruker APEX 2 DUO with microfocus X-ray generator. The structures were solved and refined using the programs SHELXS-97 and SHELXL-97[2] respectively. The program X-Seed[3] was used as an interface to the SHELX programs. The positions of the hydrogen atoms where constrained to the default values SHELX for the preparation of the crystallographic information file (.cif) but corrected to

the neutron normalized values for the structure analysis. Residual electron density found within the solvent accessible volume was removed with PLATON SQUEEZE,[4] and the crystal information file was prepared with PLATON CALCFCF-SQ.[5]

Crystallographic data: Details of X-ray crystal structure determination are available at Cambridge Crystallographic Data Centre (CCDC). The deposition number is **CCDC No 1409618;** Formula = $C_{111}H_{81}Br_{2}F_{18}N_{15}O_{22}Zn_{5}$, M = 2805.57, colourless needle, 0.100 × 0.010 × 0.010 mm^3, Trigonal, space group P-3 (No. 147), V = 7555(3) Å^3, Z = 2, D_c = 1.233 g/cm^3, F_{000} = 2820, CuKα radiation, λ = 1.54178 Å, T = 100(2) K, $2\theta_{max}$ = 116.6º, 36252 reflections collected, 6994 unique (R_{int} = 0.0935). Final GoF = 1.067, R_1 = 0.0933, wR_2 = 0.2711, R indices based on 4289 reflections with I > 2 σ(I) (refinement on F^2), 520 parameters, 0 restraints. Lp and absorption corrections applied, μ = 2.206 mm^{-1}.

checkCIF/PLATON report

Structure factors have been supplied for datablock(s) C__sadsq_sq_d

THIS REPORT IS FOR GUIDANCE ONLY. IF USED AS PART OF A REVIEW PROCEDURE FOR PUBLICATION, IT SHOULD NOT REPLACE THE EXPERTISE OF AN EXPERIENCED CRYSTALLOGRAPHIC REFEREE.

No syntax errors found.   CIF dictionary   Interpreting this report

Datablock: C__sadsq_sq_d

Bond precision: C-C = 0.0158 A   Wavelength=1.54178

Cell:	a=23.023(4)	b=23.023(4)	c=16.459(3)
	alpha=90	beta=90	gamma=120
Temperature:	100 K		

	Calculated	Reported
Volume	7555(3)	7555(3)
Space group	P -3	P -3
Hall group	-P 3	-P 3
Moiety formula	C99 H81 N15 O9 Zn3, 2(C6 Br F9 O6 Zn), O	?
Sum formula	C111 H81 Br2 F18 N15 O22 Zn5	C111 H81 Br2 F18 N15 O22 Zn5
Mr	2805.66	2805.57
Dx,g cm-3	1.233	1.233
Z	2	2
Mu (mm-1)	2.206	2.206
F000	2820.0	2820.0
F000'	2811.02	
h,k,lmax	25,25,18	25,25,18
Nref	7106	6994
Tmin,Tmax	0.974,0.978	0.649,0.753
Tmin'	0.802	

Correction method= # Reported T Limits: Tmin=0.649 Tmax=0.753
AbsCorr = MULTI-SCAN

Data completeness= 0.984   Theta(max)= 58.297

R(reflections)= 0.0933(4289)   wR2(reflections)= 0.2957(6994)

S = 1.067   Npar= 520

The following ALERTS were generated. Each ALERT has the format
        test-name_ALERT_alert-type_alert-level.
Click on the hyperlinks for more details of the test.

Alert level A

```
PLAT430_ALERT_2_A Short Inter D...A Contact  O3A    ..  O6      ..        2.39 Ang.
PLAT430_ALERT_2_A Short Inter D...A Contact  O3B    ..  O6      ..        2.33 Ang.
PLAT602_ALERT_2_A VERY LARGE Solvent Accessible VOID(S) in Structure         ! Info
```

Alert level B

```
THETM01_ALERT_3_B  The value of sine(theta_max)/wavelength is less than 0.575
            Calculated sin(theta_max)/wavelength =     0.5518
PLAT018_ALERT_1_B  _diffrn_measured_fraction_theta_max .NE. _full            ! Check
PLAT306_ALERT_2_B Isolated Oxygen Atom (H-atoms Missing ?) .......          O6 Check
PLAT341_ALERT_3_B Low Bond Precision on  C-C Bonds ..............       0.0158 Ang.
```

Alert level C

```
RFACR01_ALERT_3_C  The value of the weighted R factor is > 0.25
            Weighted R factor given    0.296
PLAT084_ALERT_3_C High wR2 Value (i.e. > 0.25) ..................         0.30 Report
PLAT094_ALERT_2_C Ratio of Maximum / Minimum Residual Density ....        2.22 Report
PLAT213_ALERT_2_C Atom F6A             has ADP max/min Ratio .....         3.1 prolat
PLAT230_ALERT_2_C Hirshfeld Test Diff for    O3A    --  C2A     ..         5.2 su
PLAT234_ALERT_4_C Large Hirshfeld Difference N27    --  C26     ..        0.16 Ang.
PLAT234_ALERT_4_C Large Hirshfeld Difference C18    --  C19     ..        0.16 Ang.
PLAT234_ALERT_4_C Large Hirshfeld Difference C19    --  C20     ..        0.16 Ang.
PLAT234_ALERT_4_C Large Hirshfeld Difference C20    --  C23     ..        0.16 Ang.
PLAT234_ALERT_4_C Large Hirshfeld Difference C35    --  C36     ..        0.20 Ang.
PLAT234_ALERT_4_C Large Hirshfeld Difference C35    --  C40     ..        0.20 Ang.
PLAT234_ALERT_4_C Large Hirshfeld Difference F7A    --  C4A     ..        0.21 Ang.
PLAT241_ALERT_2_C High      Ueq as Compared to Neighbors for .....         C23 Check
PLAT241_ALERT_2_C High      Ueq as Compared to Neighbors for .....         C34 Check
PLAT241_ALERT_2_C High      Ueq as Compared to Neighbors for .....         C36 Check
PLAT241_ALERT_2_C High      Ueq as Compared to Neighbors for .....         C37 Check
PLAT241_ALERT_2_C High      Ueq as Compared to Neighbors for .....         O1B Check
PLAT242_ALERT_2_C Low       Ueq as Compared to Neighbors for .....         C20 Check
PLAT242_ALERT_2_C Low       Ueq as Compared to Neighbors for .....         C35 Check
PLAT242_ALERT_2_C Low       Ueq as Compared to Neighbors for .....         C38 Check
PLAT242_ALERT_2_C Low       Ueq as Compared to Neighbors for .....         Zn5 Check
PLAT242_ALERT_2_C Low       Ueq as Compared to Neighbors for .....         C2B Check
PLAT250_ALERT_2_C Large U3/U1 Ratio for Average U(i,j) Tensor ....         2.4 Note
PLAT334_ALERT_2_C Small Average Benzene  C-C Dist. C17    -C22            1.36 Ang.
PLAT334_ALERT_2_C Small Average Benzene  C-C Dist. C35    -C40            1.37 Ang.
PLAT906_ALERT_3_C Large K value in the Analysis of Variance ......       8.300 Check
PLAT911_ALERT_3_C Missing # FCF Refl Between THmin & STh/L=  0.552         108 Report
PLAT913_ALERT_3_C Missing # of Very Strong Reflections in FCF ....           1 Note
PLAT918_ALERT_3_C Reflection(s) with I(obs) much smaller I(calc) .           1 Check
PLAT971_ALERT_2_C Check Calcd Residual Density  0.18A From      O6        1.55 eA-3
```

Alert level G

```
PLAT072_ALERT_2_G SHELXL First  Parameter in WGHT Unusually Large.        0.19 Report
PLAT232_ALERT_2_G Hirshfeld Test Diff (M-X)  Br4    --  Zn5     ..         8.1 su
PLAT242_ALERT_2_G Low       Ueq as Compared to Neighbors for .....         C4A Check
PLAT242_ALERT_2_G Low       Ueq as Compared to Neighbors for .....         C4B Check
PLAT304_ALERT_4_G Non-Integer Number of Atoms (   0.33) in Resd. #           4 Check
PLAT790_ALERT_4_G Centre of Gravity not Within Unit Cell: Resd.  #           4 Note
              O
```

```
PLAT910_ALERT_3_G Missing # of FCF Reflection(s) Below Th(Min) ...         1 Report
PLAT961_ALERT_5_G Dataset Contains no Negative Intensities .......     Please Check
```

```
  3 ALERT level A = Most likely a serious problem - resolve or explain
  4 ALERT level B = A potentially serious problem, consider carefully
 30 ALERT level C = Check. Ensure it is not caused by an omission or oversight
  8 ALERT level G = General information/check it is not something unexpected

  1 ALERT type 1 CIF construction/syntax error, inconsistent or missing data
 25 ALERT type 2 Indicator that the structure model may be wrong or deficient
  9 ALERT type 3 Indicator that the structure quality may be low
  9 ALERT type 4 Improvement, methodology, query or suggestion
  1 ALERT type 5 Informative message, check
```

It is advisable to attempt to resolve as many as possible of the alerts in all categories. Often the minor alerts point to easily fixed oversights, errors and omissions in your CIF or refinement strategy, so attention to these fine details can be worthwhile. In order to resolve some of the more serious problems it may be necessary to carry out additional measurements or structure refinements. However, the purpose of your study may justify the reported deviations and the more serious of these should normally be commented upon in the discussion or experimental section of a paper or in the "special_details" fields of the CIF. checkCIF was carefully designed to identify outliers and unusual parameters, but every test has its limitations and alerts that are not important in a particular case may appear. Conversely, the absence of alerts does not guarantee there are no aspects of the results needing attention. It is up to the individual to critically assess their own results and, if necessary, seek expert advice.

Publication of your CIF in IUCr journals

A basic structural check has been run on your CIF. These basic checks will be run on all CIFs submitted for publication in IUCr journals (*Acta Crystallographica*, *Journal of Applied Crystallography*, *Journal of Synchrotron Radiation*); however, if you intend to submit to *Acta Crystallographica Section C* or *E*, you should make sure that full publication checks are run on the final version of your CIF prior to submission.

Publication of your CIF in other journals

Please refer to the *Notes for Authors* of the relevant journal for any special instructions relating to CIF submission.

PLATON version of 21/06/2015; check.def file version of 21/06/2015

Datablock C__sadsq_sq_d - ellipsoid plot

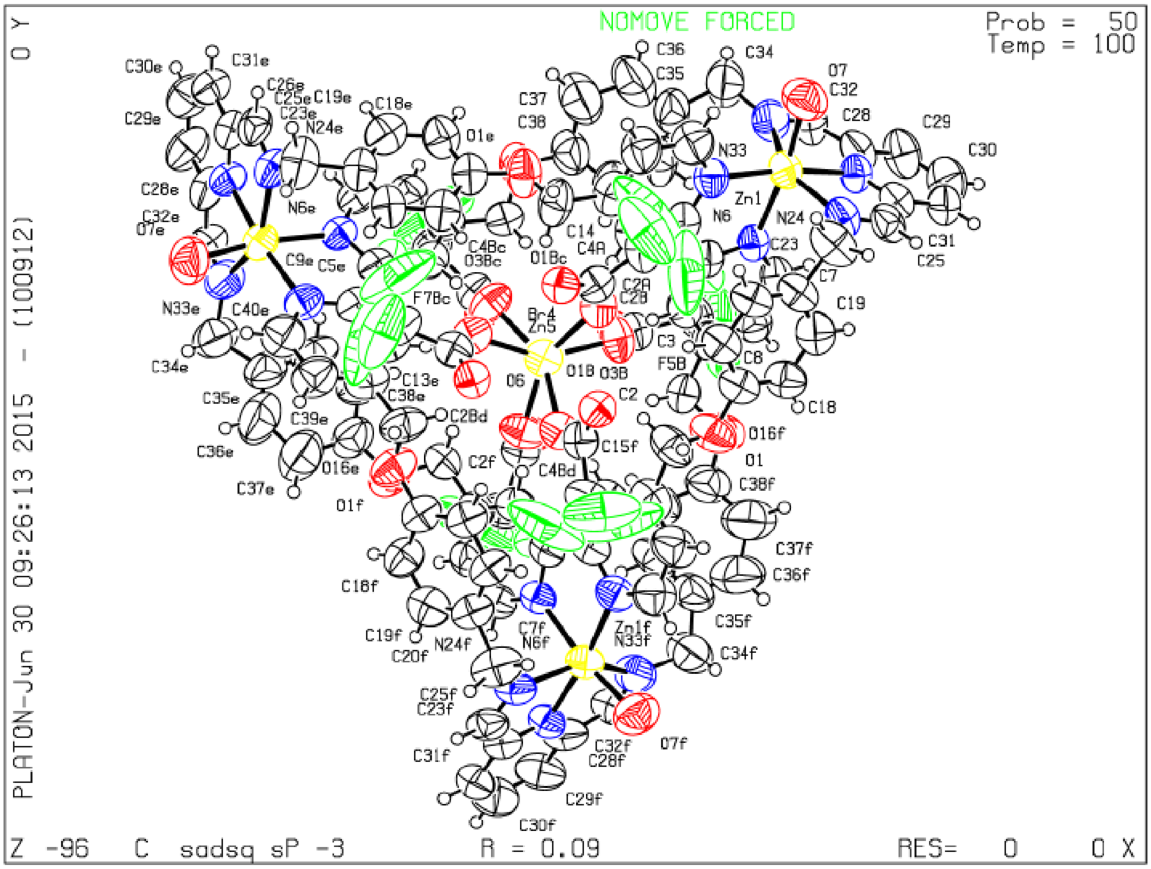

The single crystals of TKs diffract weakly at high angle as shown by the low data completeness (about 98%). This prevents a complete structure refinement and explains the moiety formula, which refers to the part of the structure that could be refined. The counter ion(s) that balance the charge are likely to be sitting in the voids within the trefoil knots. This explains the A alert in the checkCIF. We also note a relatively short interatomic distance between O3 and O6 of about 2.4 Å. This is not surprising considering the geometry of the interactions with 2 sets of three charged carbonyl species.

The same crystal used for single crystal XRD characterisation was placed on a polymeric support and analysed by EDAX to confirm the presence of bromide anions, EDAX analysis is presented in Figure S1b.

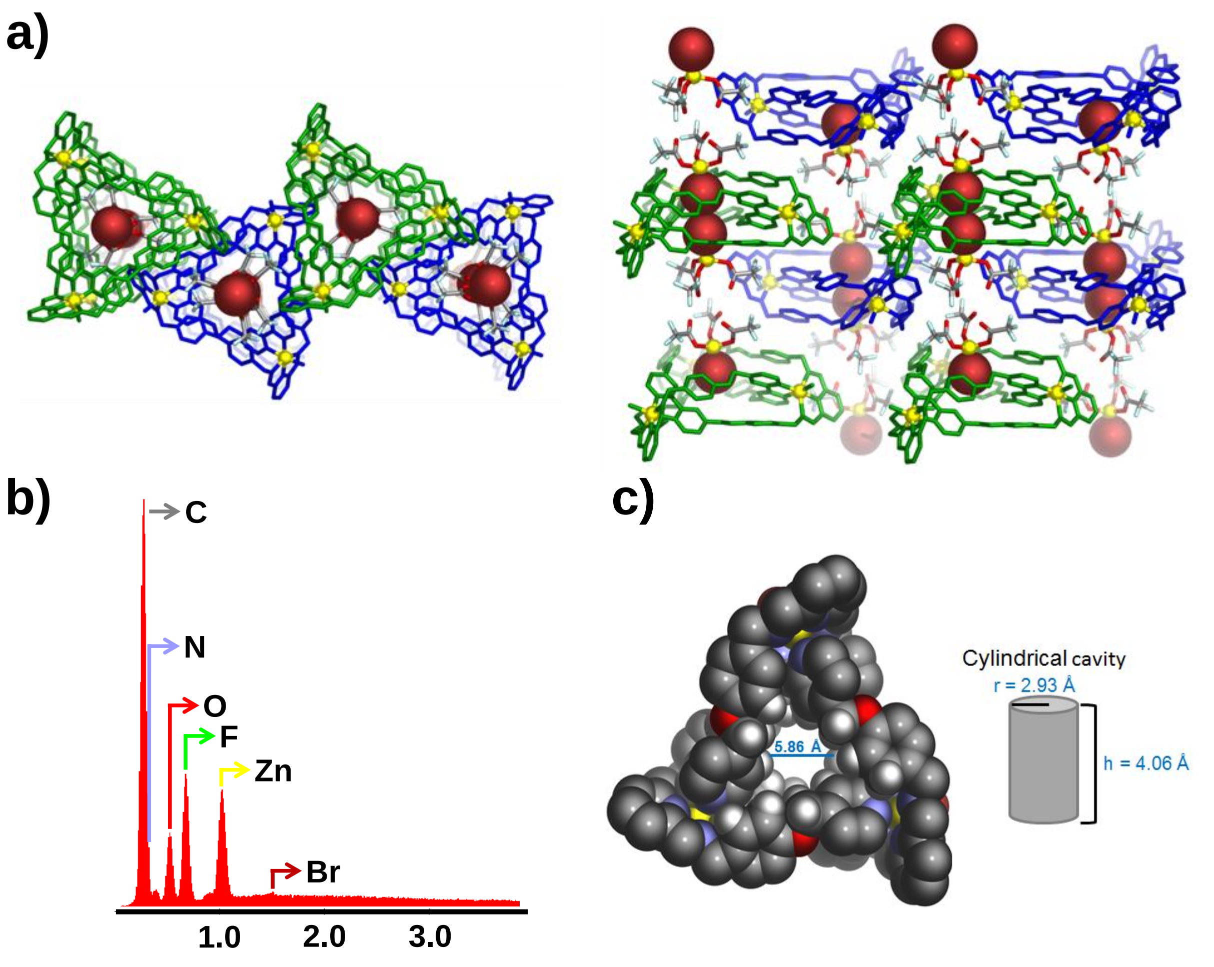

Figure S1: a) Crystal packing of **TK**$^{6+}$ and the adduct $[ZnBr(CF_3COO)_3]^{2-}$. b) EDAX analysis of a single **TK**$^{6+}$'s crystal.

3.0 Theoretical calculations

Full geometry optimizations of **TK**$^{6+}$, $[$**TK**$(X)_2]^{4+}$ (X = Br^-, SCN^-, NO_3^- or BF_4^-), **SL**$^{8+}$ and $[$**SL**$(Otf)]^{7+}$ systems were carried out at the PM6 semi-empirical level with the Gaussian 09 program package.[6] No symmetry constraints were imposed. Stationary points found on the potential energy surfaces were tested via frequency analysis (no imaginary frequencies) to assure

that they represented energy minima rather than saddle points. The electrostatic potential of **TK**$^{6+}$ was computed at the B3LYP/6-31G(d) level[7,8] (the smaller 3-21G basis set was selected for Zn) using the PM6 optimized geometry.

The Cartesian coordinates of the optimized geometries can be provided upon request.

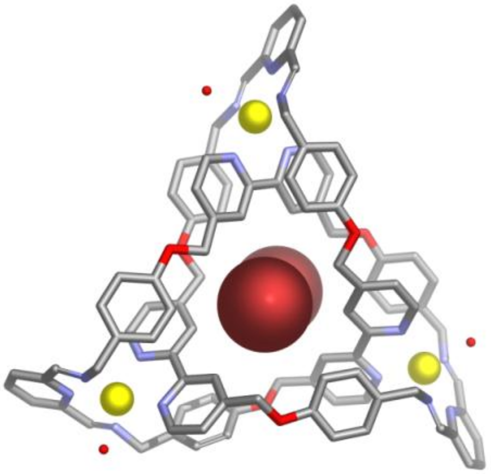

Figure S2: PM6-optimized structures of [**TK**$(Br)_2]^{4+}$ complexes.

4.0 Anion recognition studies of TK(TFA)$_6$

All titrations were carried out in D_2O at 298 K. Changes in the ^1H NMR spectral shifts of **TK**$^{6+}$ and the binding isotherms of titration with different anions are presented in figures S3 – S6. The model used to fit the NMR data is based on anion/TK 1:1 and 2:1 ratio. Attempts to fit according to a single 1:1 species failed. The data were fitted according to the method of Taylor and Anderson,[9-11] using the equation ((a*b*x+c*d*x^2)/(1+b*x+d*x^2)) to fit the data. The constants obtained were found to be in good agreement with those derived from the fitting with the WinEqNMR software.[15]

4.1 *Iodide*

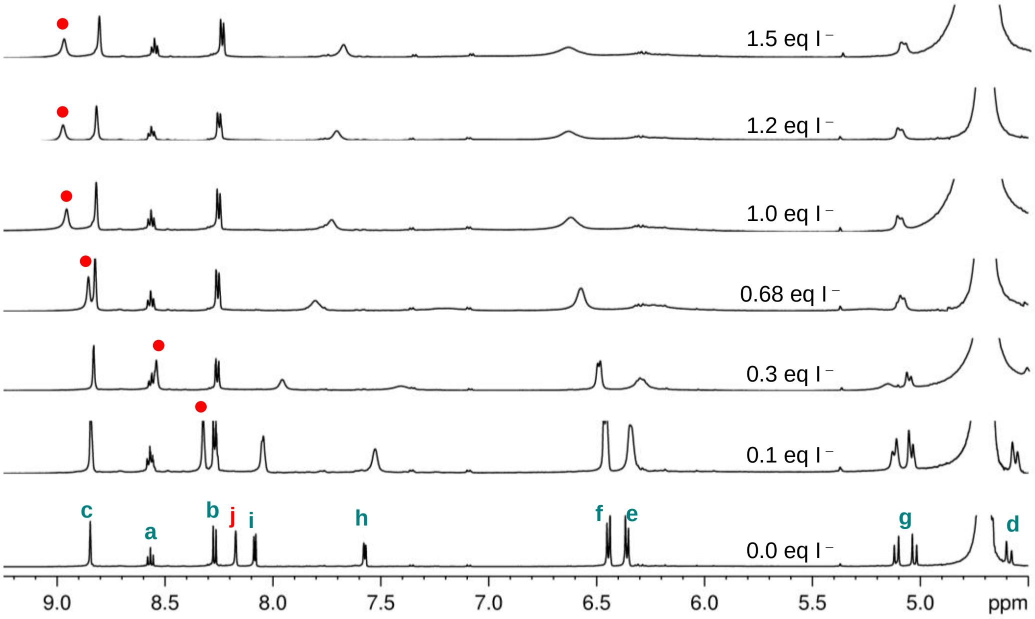

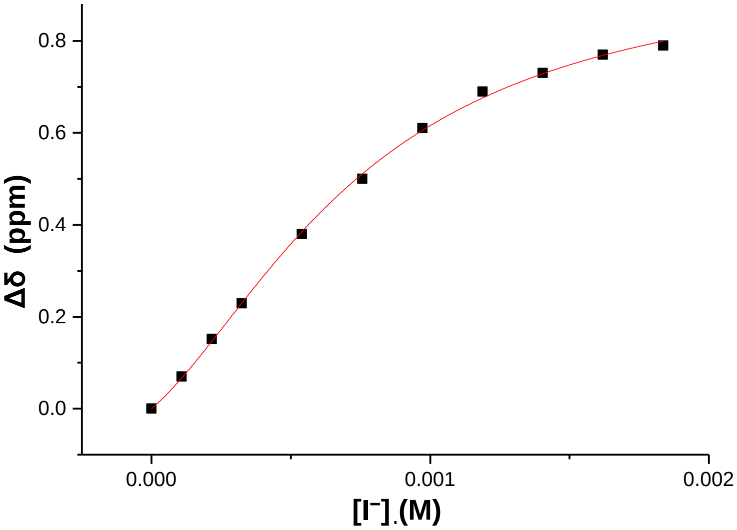

Figure S3: Change in ^{1}H NMR (600 MHz, 298 K) chemical shift (black squares) of H_j protons and calculated binding isotherm (red curve) obtained by titrating a solution of **TK**(TFA)$_6$ in D_2O (1.87 mM) with an aqueous solution of tetramethylammonium iodide (0.054 M) (data for H_j protons).

4.2 *Azide*

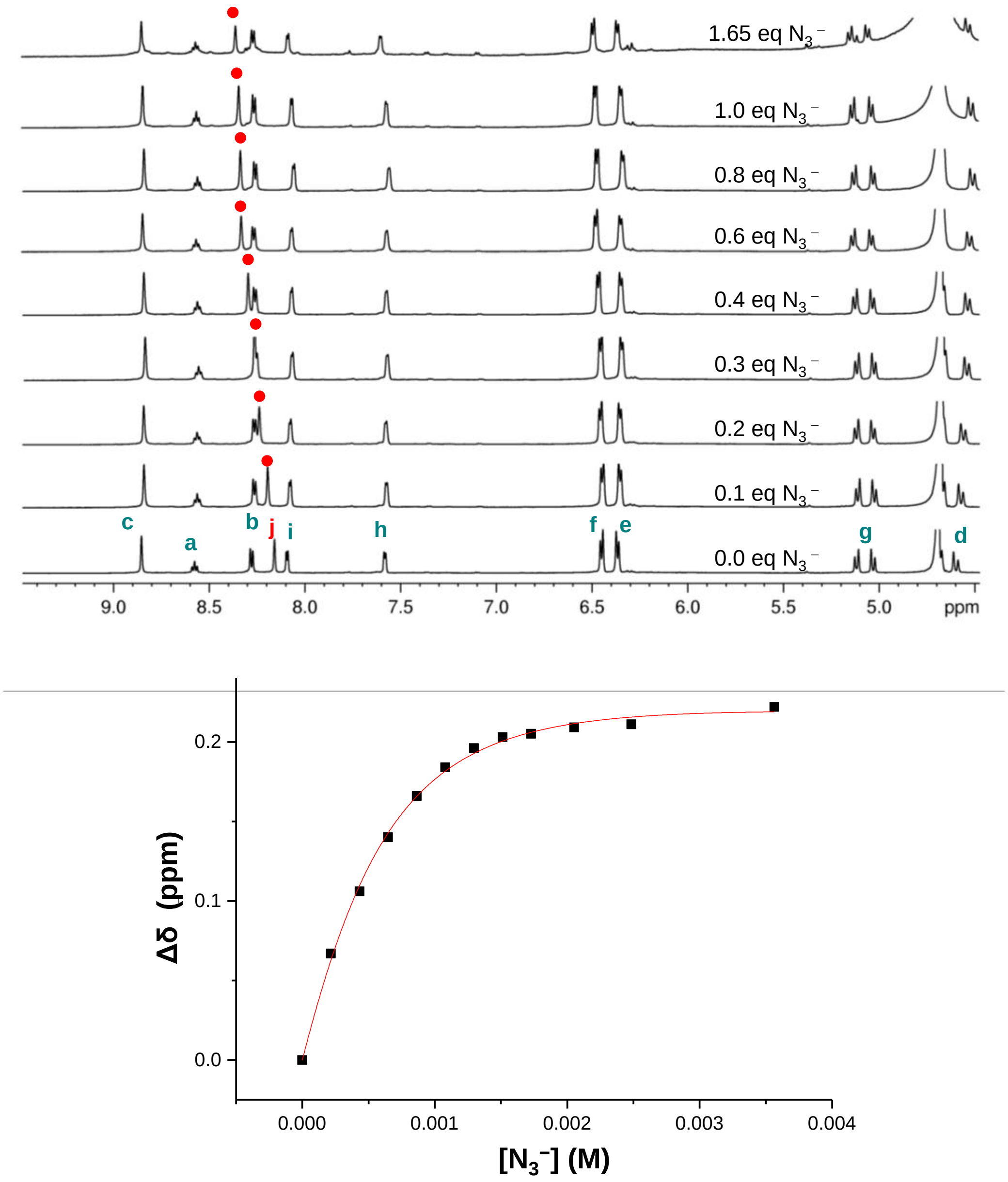

Figure S4: Change in ^{1}H NMR (600 MHz, 298 K) chemical shift (black squares) of H_j protons and calculated binding isotherm (red curve) obtained by titrating a solution of **TK**(TFA)$_6$ in D_2O (1.87 mM) with an aqueous solution of sodium azide (0.054 M) (data for H_j protons).

4.3 *Thiocycanate*

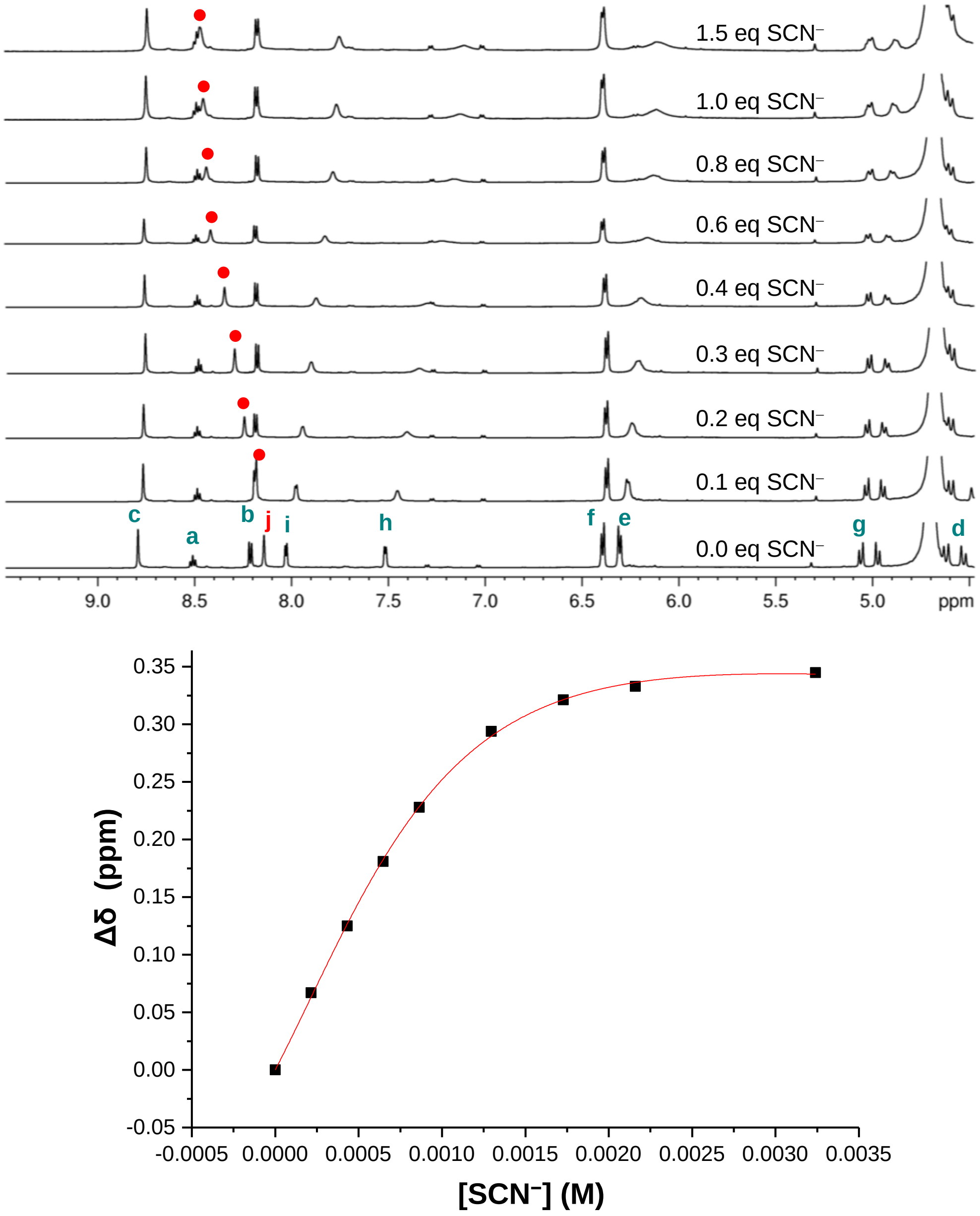

Figure S5: Change in ^{1}H NMR (600 MHz, 298 K) chemical shift (black squares) of H_j protons and calculated binding isotherm (red curve) obtained by titrating a solution of **TK**$(TFA)_6$ in D_2O (1.87 mM) with an aqueous solution of ammonium thiocyanate (0.054 M) (data for Hj protons).

4.4 *Nitrate*

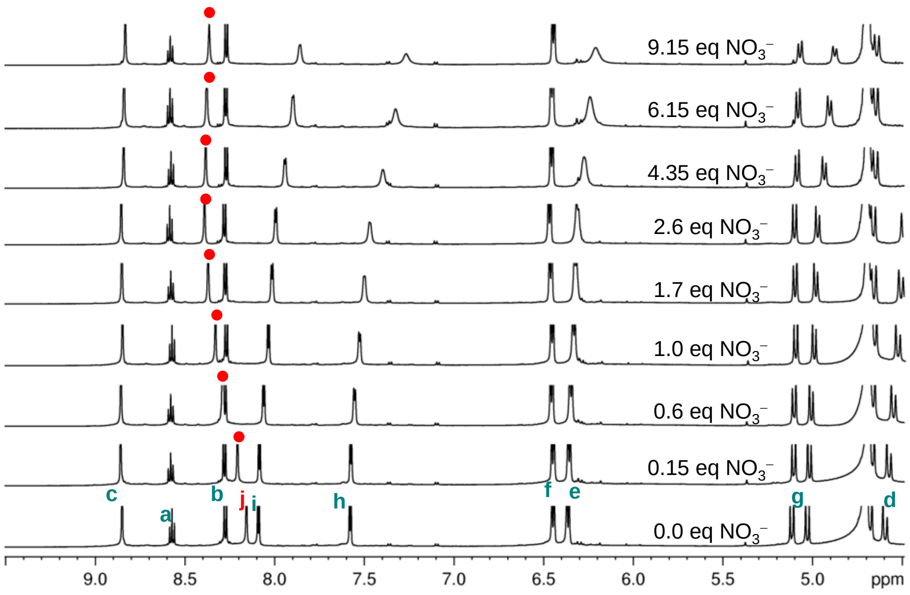

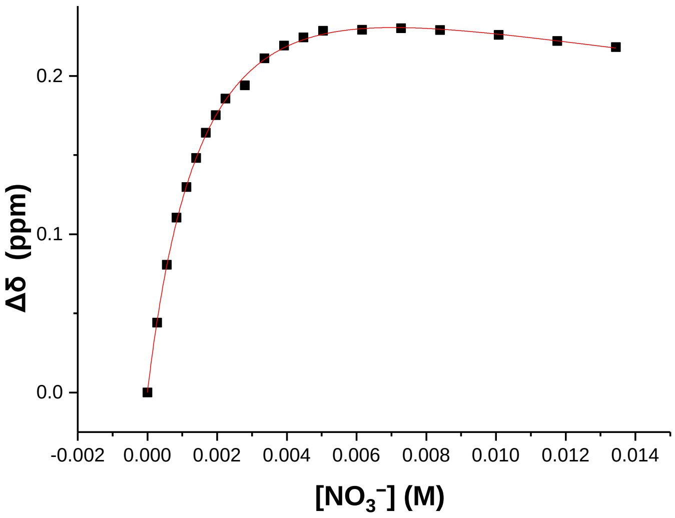

Figure S6: Change in ^{1}H NMR (600 MHz, 298 K) chemical shift (black squares) of H_j protons and calculated binding isotherm (red curve) obtained by titrating a solution of **TK**$(TFA)_6$ in D_2O (1.87 mM) with an aqueous solution of tetrabutylammonium nitrate (0.056 M) (data for H_j protons).

4.5 *Tetrafluoroborate*

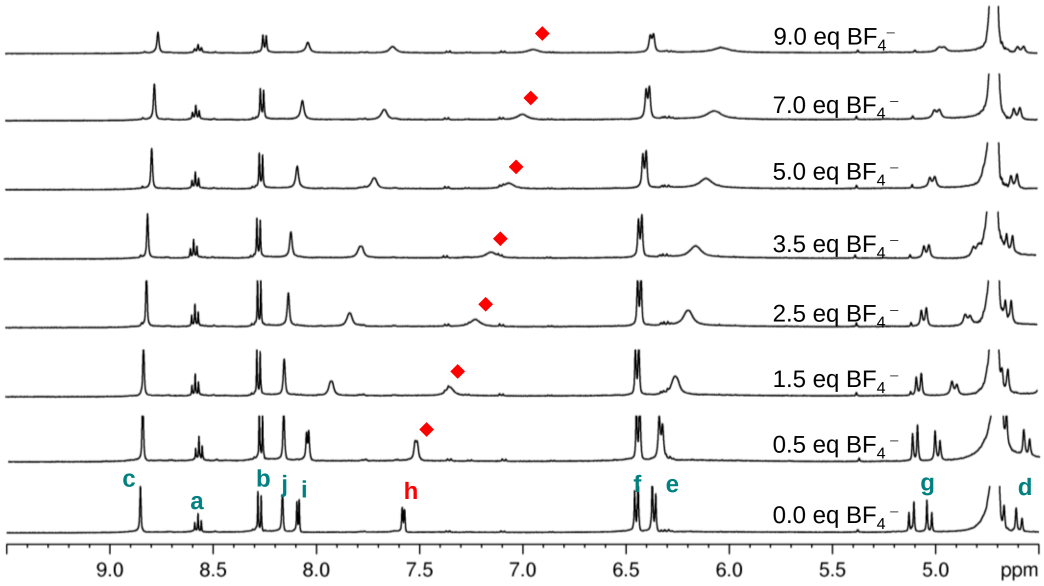

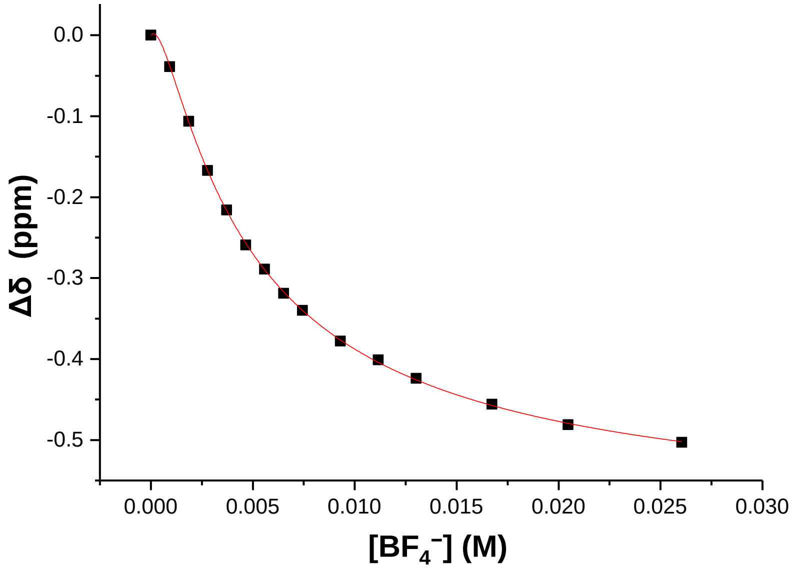

Figure S7: Change in ^{1}H NMR (600 MHz, 298 K) chemical shift (black squares) of H_h protons and calculated binding isotherm obtained by titrating a solution of **TK**(TFA)$_6$ in D_2O (1.87 mM) with an aqueous solution of tetramethylammonium tetrafluoroborate (0.093 M) (data for H_h protons).

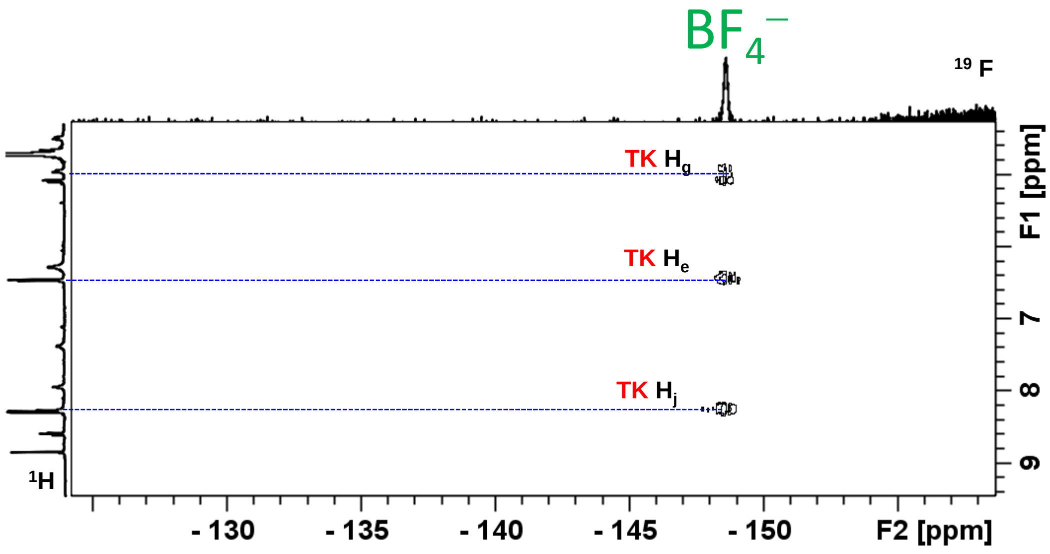

Figure S8: HOESY spectrum at 500 MHz and 298 K of D_2O solution of tetramethylammonium tetrafluoroborate and **TK**$(TFA)_6$.

4.6 *Trifluoromethanesulfonate*

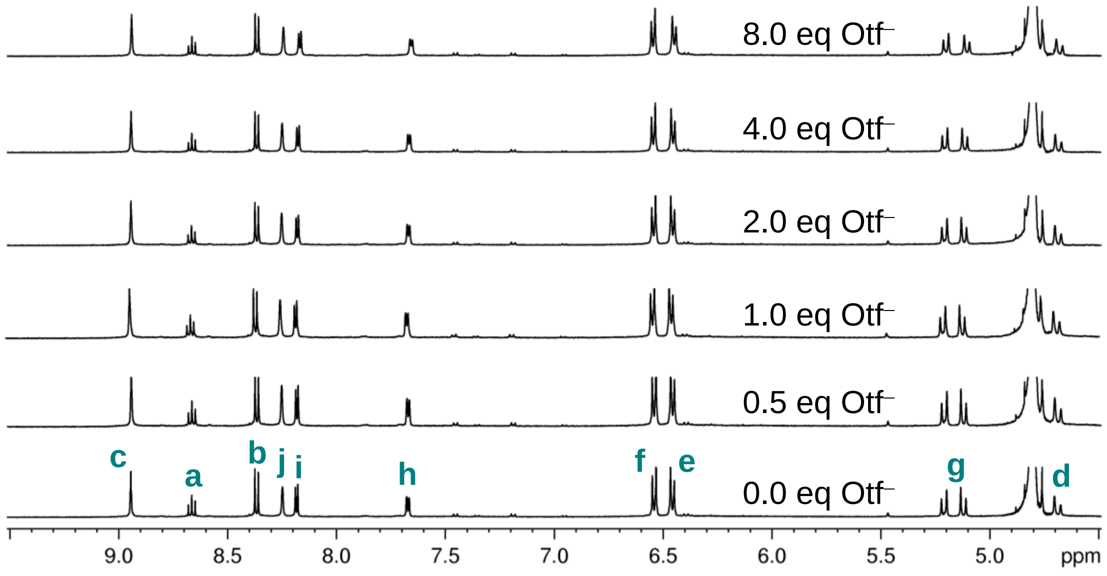

Figure S9: ^{1}H NMR (600 MHz, 298 K) spectra associated with the titration of a solution of **TK**$(TFA)_6$ in D_2O (1.87 mM) with an aqueous solution of ammonium trifluoromethanesulfonate (0.0938 M). No significant changes in the chemical shifts of the **TK**$(TFA)_6$ resonances were observed.

4.7 HRMS-ESI of a 1 : 2 mixture of TK : Anion in water

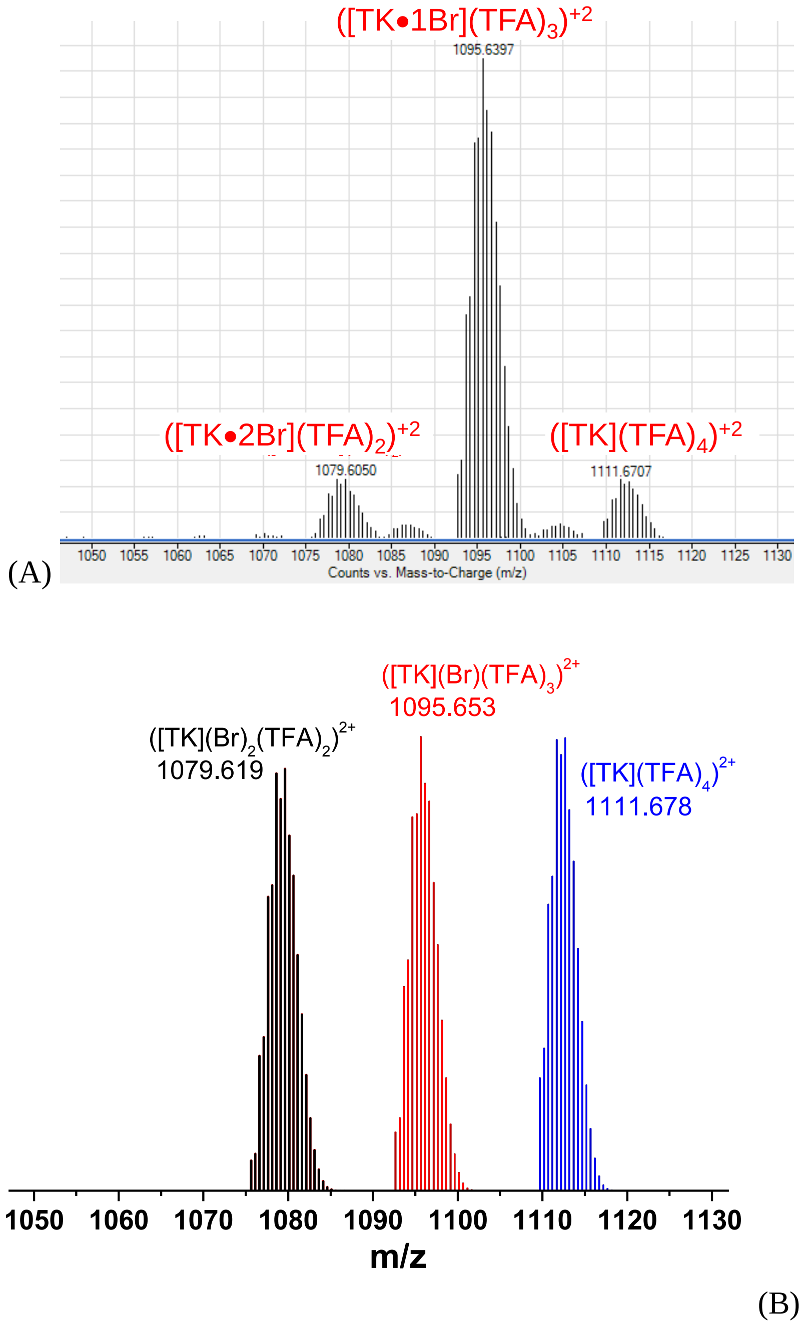

Figure S10: Zoom into the +2 charged clusters of the 1: 2 mixture of **TK** : TBABr. The clusters correspond to ([**TK**•2Br]$(TFA)_2)^{+2,}$ ([**TK**•1Br]$(TFA)_3)^{+2}$ and ([**TK**]$(TFA)_4)^{+2}$ obtained by HRMS-ESI. Experimental data (top), simulated data (bottom).

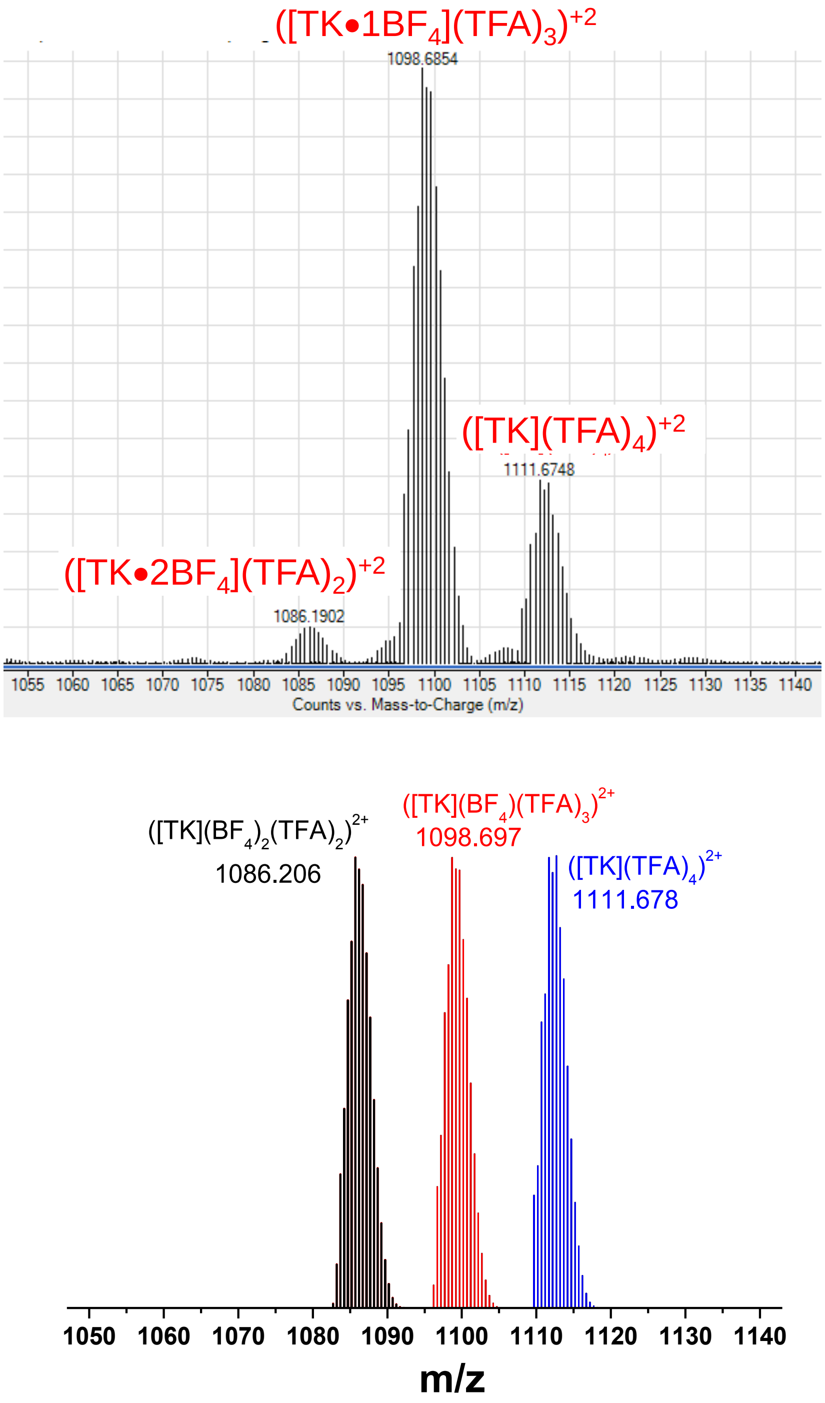

Figure S11: Zoom into the +2 charged clusters of the 1: 2 mixture of TK : $TMABF_4^-$. The clusters correspond to ([**TK**•$2BF_4$]$(TFA)_2$)$^{+2,}$ ([**TK**•$1BF_4$]$(TFA)_3$)$^{+2}$ and [**TK**$(TFA)_4$]$^{+2}$ obtained by HRMS-ESI. Experimental data (top) and simulated data (bottom).

4.8 Speciation diagrams of the host-guest complexes of TK with various anions

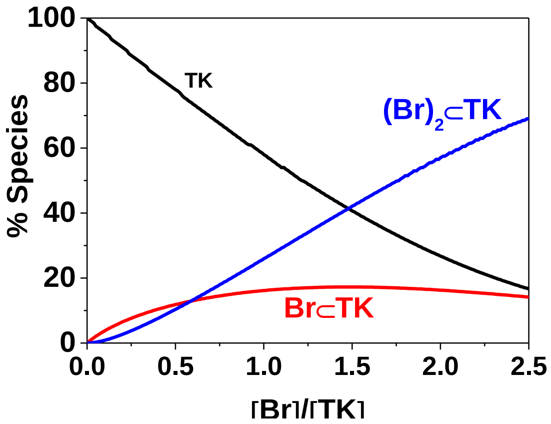

Figure S12: Speciation diagrams of the host-guest complexes of TK with NBu_4Br. Solvent: water; T = 25°C; [TK] = 2 mM.

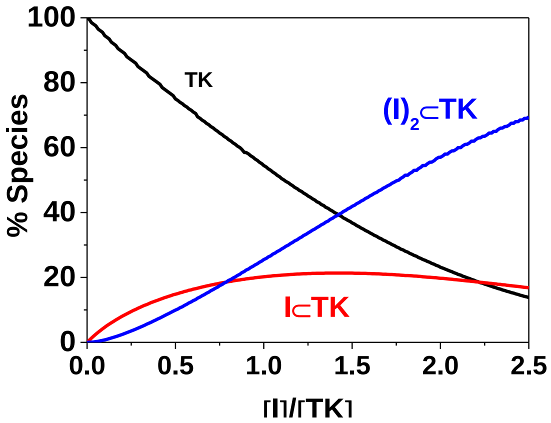

Figure S13: Speciation diagrams of the host-guest complexes of TK with NBu_4I. Solvent: water; T = 25°C; [TK] = 2 mM.

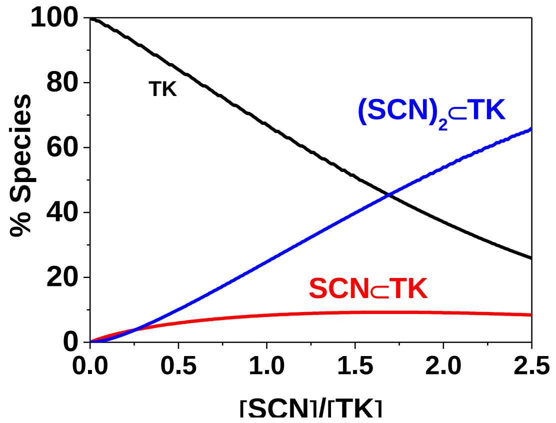

Figure S14: Speciation diagrams of the host-guest complexes of TK with $NBu_4(SCN)$. Solvent: water; T = 25°C; [TK] = 2 mM.

4.9 Variable temperature ^1H NMR of TK(TFA)6 in presence of Br^- and BF_4^- and ^{19}F – ^1H HOESY NMR of $TK(TFA)_6$

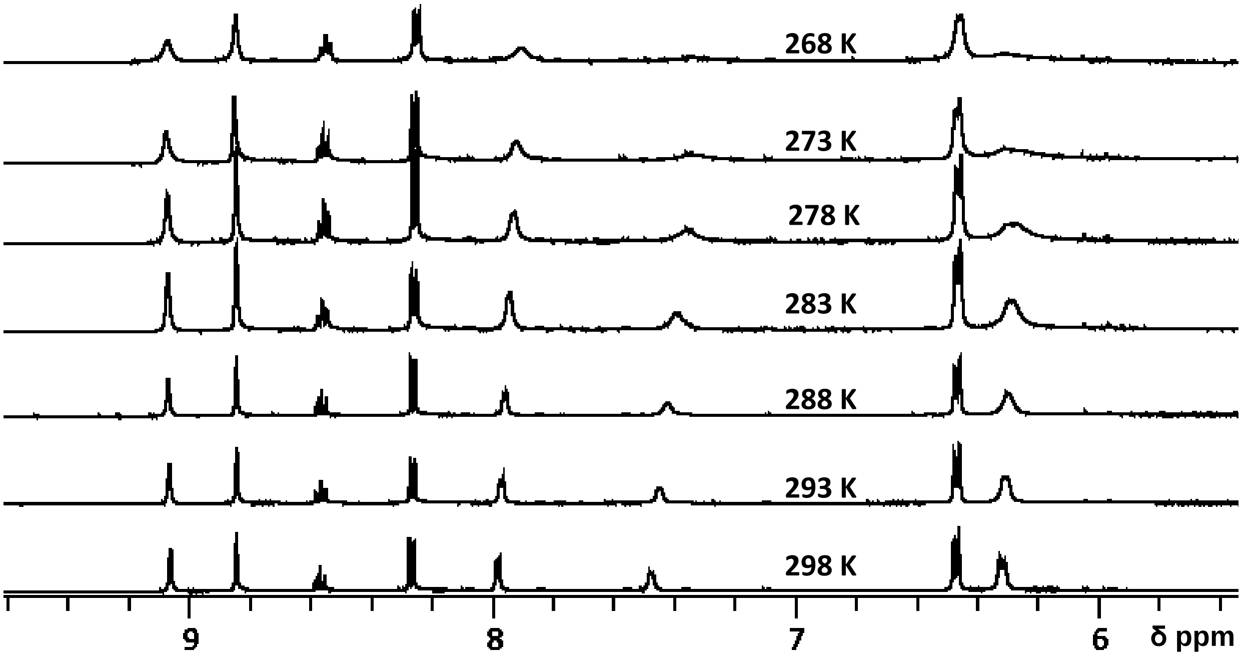

Figure S15: Variable temperature of ^1H NMR spectra host-guest complexes of **TK**$(TFA)_6$ with 2.5 equivalent of tetrabutylammonium bromide in D_2O

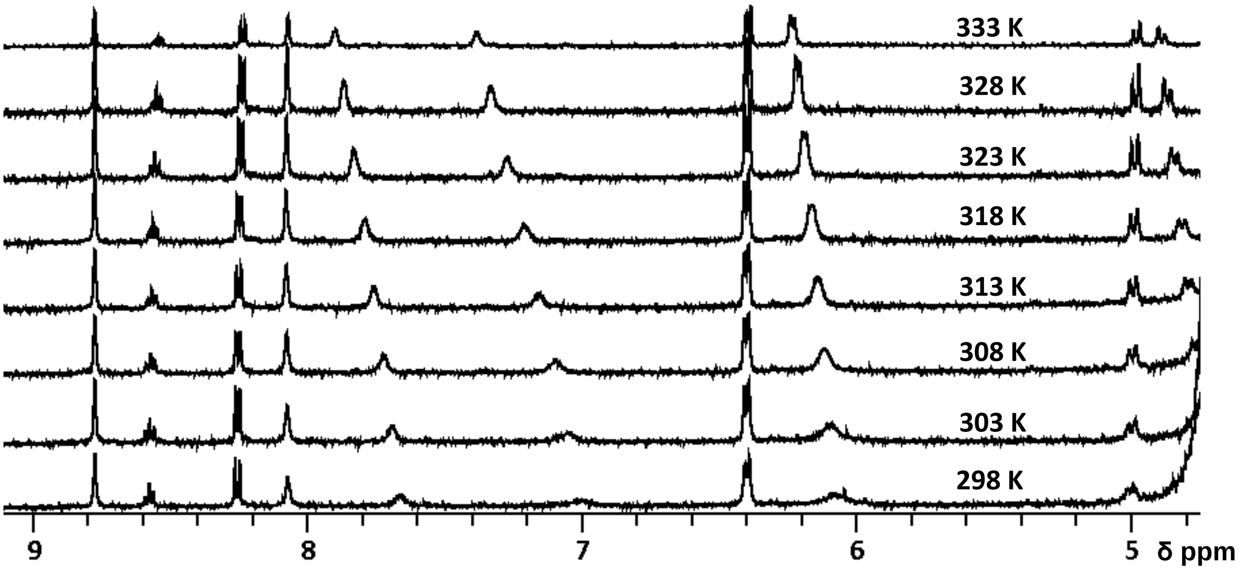

Figure S16: Variable temperature of ^{1}H NMR spectra host-guest complexes of TK with 9.0 equivalent of tetramethylammonium tetrafluoroborate in D_2O

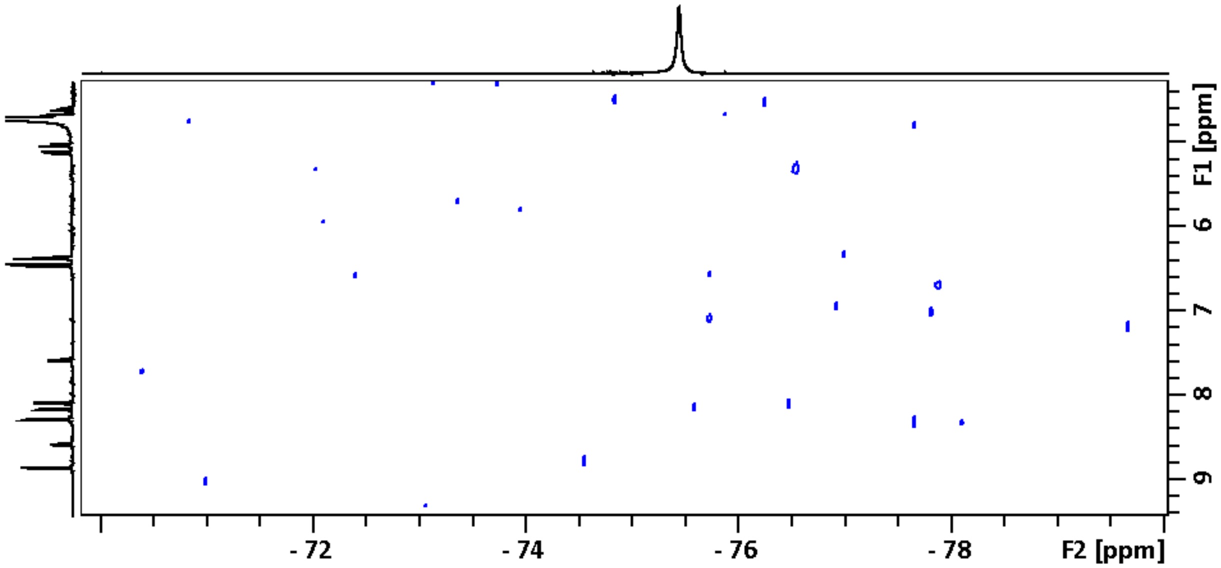

Figure S17: ^{19}F – ^{1}H HOESY NMR of **TK**$(TFA)_6$ in D_2O at room temperature.

5.0 Synthesis of neutral DAB

Trifluoroacetate salt of DAB was prepared according to our previously published procedure.[1] The trifluoroacetate salt of DAB was dissolved in water and neutralized through a drop wise addition of a 1 M NaOH solution till the ligand completely precipitate out of solution. After neutralization, the precipitate was filtered and dried under vacuum for 4h. Yield: 98 %; ^{1}H NMR (600 MHz, CD_3OD-d_4, 25 °C): δ 3.72 (s, 4H,Ar-C*H₂*), 5.24 (s, 4H,Ar-C*H₂*), 7.01 (d, 4H, *J* = 7.9 Hz, Ar-*H*), 7.27 (d, 4H, *J* = 7.9 Hz, Ar-*H*), 7.52 (d, 2H, *J* = 4.3 Hz, Ar-*H*), 8.41 (s, 2H, Ar-*H*), 8.64 (d, 2H, *J* = 4.7 Hz, Ar-*H*); ^{13}C NMR (600 MHz, CD_3OD-d_4, 25 °C): δ 44.7, 67.8, 114.6, 119.1, 121.8, 128.4, 135.2, 148.7, 149.0, 155.8, 157.3; MS (ESI-HRMS): m/z Calculated for $C_{26}H_{27}N_4O_2$: 427.2129 $[M+H]^+$, found: 427.2125 $[M+H]^+$.

6.0 Controlling the population of [2]C^{4+}, TK^{6+}, and SL^{8+} in solution

6.1 *[2]Catenane*

Four reactions having the same the contents were heated to different temperatures. In each, the neutral DAB (6 mg, 0.014 mmol) was stirred with zinc acetate $Zn(OAc)_2$ (3.84 mg, 0.02 mmol) and diformyl pyridine (DFP) (2 mg, 0.014 mmol) in a mixture of 0.3 mL D_2O and 0.3 mL CD_3OD. The reactions were heated to 323, 338, 348 or 363 K, and the ^{1}H NMR spectrum of each mixture was recorded 24 h after preparation. The relative amounts of the components of the reaction mixtures were determined by signal integration. The reactions maintained at 323, 338 or 348 K resulted in the formation of mixtures of **[2]C** and **TK**, whereas the reaction maintained at 363 K resulted in the exclusive formation of **[2]C**, though some minor peaks corresponding to starting materials were present in the corresponding spectrum.

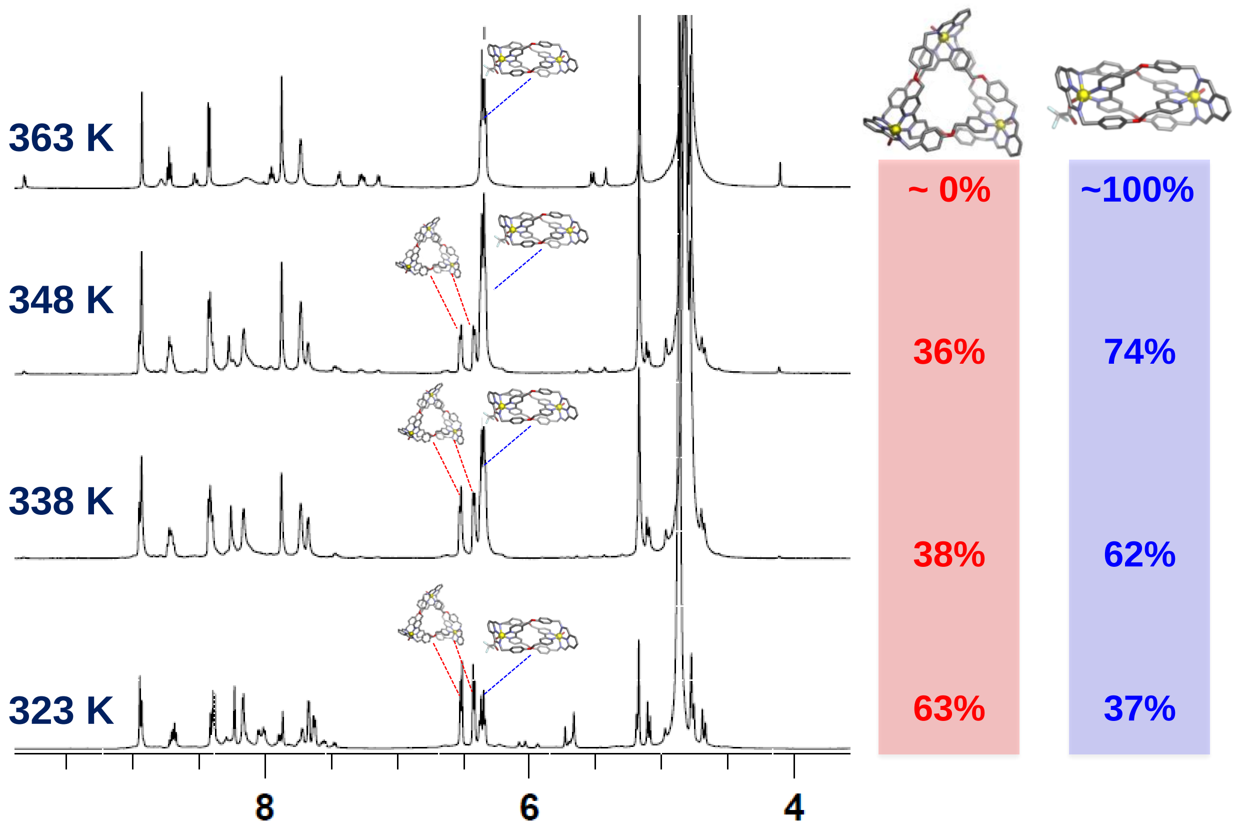

Figure S18: ^{1}H NMR spectra (500 MHz and 298 K) of reaction mixtures involving the templation of **TK**$^{6+}$ in solution, with tetrabutylammonium bromide used as the source of bromide.

.

6.2 *Trefoil knot*

To investigate the templation effect of bromide on the formation of **TK** in solution, three reactions (a-c) with same content or the starting material but different amounts of bromide were prepared. In all three reactions, neutral DAB (6 mg, 0.014 mmol) was stirred with zinc acetate $Zn(OAc)_2$ (3.84 mg, 0.02 mmol) and diformyl pyridine (DFP) (2 mg, 0.014 mmol) in 0.3 : 0.3 mL D_2O : CD_3OD. Before leaving the reactions at 323 K overnight, one equivalent (4.5 mg, 0.014 mmol) and two equivalents (9 mg, 0.028 mmol) of tetrabutylammonium bromide were added to reaction b and c respectively.

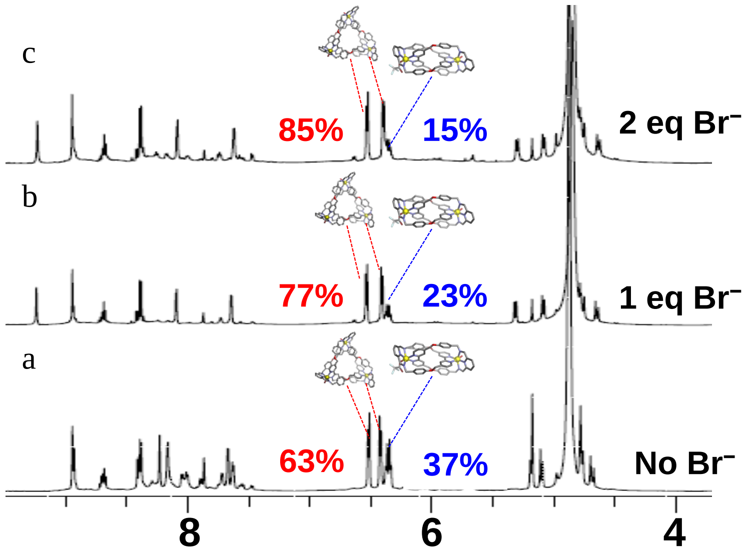

Figure S19: ^{1}H NMR spectra (500 MHz and 298 K) of the templation of the **TK**$^{6+}$ in solution using tetrabutylammonium bromide.

6.3 *Solomon link*

Solomon link was detected in solution when a larger counter ion was used in the preparation of the Zn(II) non-trivial complexes. Neutral DAB (6 mg, 0.014 mmol) was stirred with zinc triflate $Zn(OTf)_2$ (6.10 mg, 0.02 mmol) and diformyl pyridine (DFP) (2 mg, 0.014 mmol) in solvent mixture of D_2O: CD_3OD : CD_3CN (0.2: 0.2: 0.2 mL) at 323 K overnight. A mixture of all three complexes (**[2]C**, **TK** and **SL**) was obtained and characterized by NMR spectroscopy and HRMS. ^{1}H NMR (600 MHz, D_2O: CD_3OD: CD_3CN (0.2: 0.2: 0.2), 25 °C) δ: 4.97,5.00, 5.05, 5.07, 5.21, 5.23,5.27, 6.23, 6.25, 6.31, 6.40,7.54, 7.55, 7.58, 7.61,7.62, 7.74, 7.96, 8.02, 8.23, 8.25, 8.28, 8.31, 8.53, 8.55, 8.57, 8.59, 8.60, 8.61, 8.63, 8.78, 8.81, 8.12; ESI-HRMS: (*m/z*): calculated for [**SL**$(OTf)_6]^{2+}$ is 1629.16, found is 1629.13, calculated for ([**SL**$(OTf)_5]^{3+}$ is 1036.45 found is 1036.44, calculated for [**SL**$(OTf)_4]^{4+}$ is 740.10 and found is 740.09, calculated for [**SL**$(OTf)_3]^{5+}$ is 562.29 and found is 562.28.

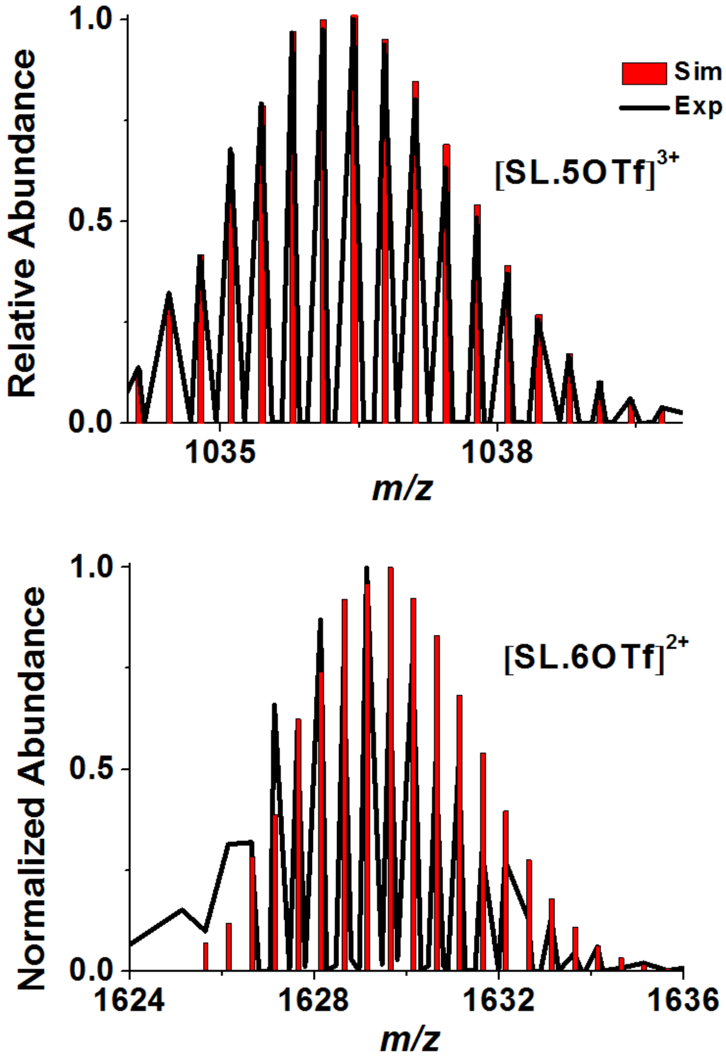

Figure S20: Experimental (Exp, black and gray traces) and simulated (Sim, blue bars) HRMS signals corresponding to the **SL(OTf)$_6$**$^{2+}$ (top) and **SL(OTf)$_5$**$^{3+}$ (bottom) ions.

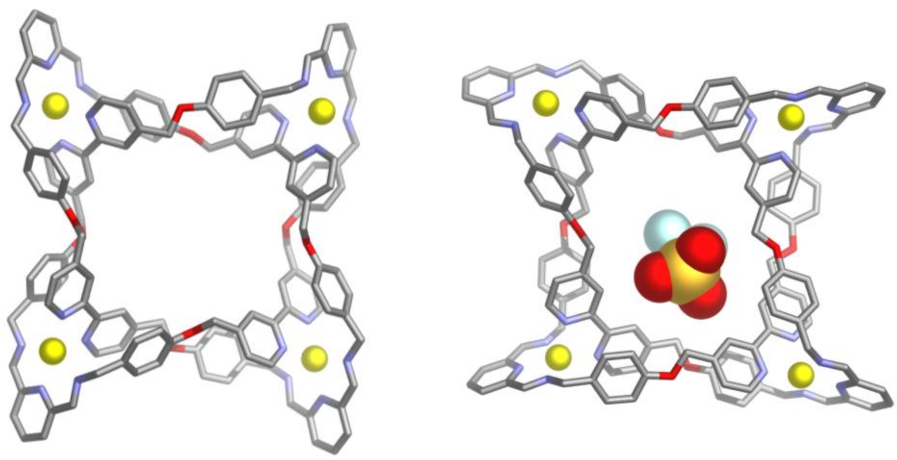

Figure S21: PM6-optimized structures of **SL**$^{8+}$ (left) and [**SL(**Otf)]$^{7+}$ complex (right).

6.4 *Hydrodynamic volume calculations*

The diffusion coefficient D is related to the hydrodynamic radius, r, of a molecule[12] by the equation:

$$D = \frac{k_B T}{6\pi\eta r} \quad (1)$$

where k_B is Boltzmann constant (1.3806488 × 10^{-23} m^2 kg s^{-2} K^{-1}), T is the absolute temperature and η the viscosity of the fluid. The solvent mixture used for the samples measured by DOSY NMR was composed of equal volumes of D_2O, CD_3CN and CD_3OD. The viscosity of this mixture, assuming ideal behavior, is given by the following equation:

$$\ln(\eta_{id}) = \sum_i x_i \, \eta_i \quad (2)$$

where x_i are the molar fractions of the different solvents.[13] Using densities of 1.11; 0.888 and 0.844 g.cm^3 respectively for D_2O, CD_3OD and CD_3CN,[14] the calculated molar fractions are respectively 0.55, 0.25 and 0.20 and the calculated viscosity is 0.818 cP (8.18×10^{-4} Pa.s).

Using equation (1), hydrodynamic radii of 1.35, 1.65 and 1.83 nm were calculated for **[2]C**, **TK** and **SL**, respectively and corresponding to hydrodynamic volumes of 10.4, 18.8 and 25.5 nm^3. The relatively small volume of **[2]C** is consistent with X-ray analysis and theoretical modeling which reveal a compact structure that lacks internal cavities.[1] The larger volume of is consistent with X-ray analysis and theoretical modeling which indicate the presence of an internal cavity. The hydrodynamic volume of **SL** is the largest of the three and is consistent with theoretical modeling which suggests that this link has the largest internal cavity.

<u>Figure 3 from the main text</u>

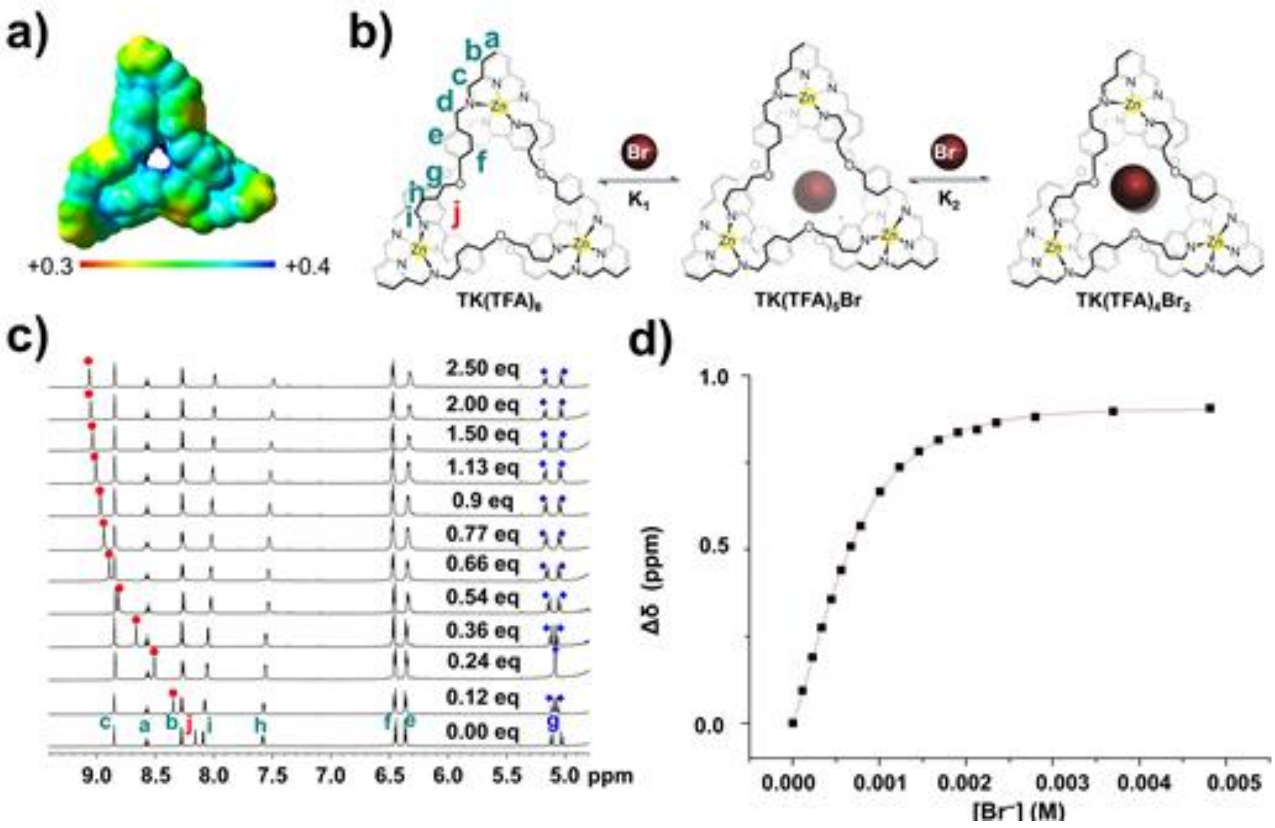

7.0 References

1 Prakasam, T. *et al.* Simultaneous self-assembly of a [2]catenane, a trefoil knot, and a solomon link from a simple pair of ligands. *Angew Chem Int Ed* **52**, 9956-9960, doi:10.1002/anie.201302425 (2013).

2 Sheldrick, G. A short history of SHELX. *Acta Cryst.* **64**, 112-122, doi:doi:10.1107/S0108767307043930 (2008).
3 Barbour, L. J. X-Seed - A software tool for supramolecular crystallography. *Supramolecular Chemistry* **1**, 189-191 (2001).
4 PLATON: A Multipurpose Crystallographic Tool (Utrecht University, Utrecht, The Netherlands, 2008).
5 Spek, A. L. Structure validation in chemical crystallography. *Acta Crystallogr D* **65**, 148-155, doi:Doi 10.1107/S090744490804362x (2009).
6 Frisch, M. *et al.* Gaussian 09, Revision A. 02, Gaussian. *Inc., Wallingford, CT* **200** (2009).
7 Becke, A. D. Density-functional thermochemistry. III. The role of exact exchange. *The Journal of chemical physics* **98**, 5648-5652 (1993).
8 Lee, C., Yang, W. & Parr, R. G. Development of the Colle-Salvetti correlation-energy formula into a functional of the electron density. *Physical Review B* **37**, 785-789 (1988).
9 Taylor, P. N. & Anderson, H. L. Cooperative Self-Assembly of Double-Strand Conjugated Porphyrin Ladders. *J. Am. Chem. Soc.* **121**, 11538-11545, doi:10.1021/ja992821d (1999).
10 Ackermann, T. K. A. Connors: Binding constants — the measurement of molecular complex stability, John Wiley & Sons, New York, Chichester, Brisbane, Toronto, Singapore 1987. 411 Seiten, Preis: £ 64.15. *Berichte der Bunsengesellschaft für physikalische Chemie* **91**, 1398-1398, doi:10.1002/bbpc.19870911223 (1987).
11 Connors, K. A. *Binding constants: the measurement of molecular complex stability*. (Wiley-Interscience, 1987).
12 Evans, R. *et al.* Quantitative Interpretation of Diffusion-Ordered NMR Spectra: Can We Rationalize Small Molecule Diffusion Coefficients? *Angew Chem In.Ed.* **52**, 3199-3202 (2013).
13 Papaioannou, D., Evangelou, T. & Panayiotou, C. Dynamic viscosity of multicomponent liquid mixtures. *J. Chem. Eng. Data* **36**, 43-46 (1991).
14 Holz, M., Mao, X. a., Seiferling, D. & Sacco, A. Experimental study of dynamic isotope effects in molecular liquids: Detection of translation-rotation coupling. *J. Chem. Phys.* **104**, 669-679 (1996).
15 Hynes, M.J., EQNMR: A computer program for the calculation of stability constants from nuclear magnetic resonance chemical shift data, J. *Chem. Soc., Dalton Trans*. 311-312 (1993).
